# Phenology analysis for trait prediction using UAVs in a MAGIC rice population with different transplanting protocols

**DOI:** 10.3389/frai.2024.1477637

**Published:** 2025-01-23

**Authors:** Shoji Taniguchi, Toshihiro Sakamoto, Haruki Nakamura, Yasunori Nonoue, Di Guan, Akari Fukuda, Hirofumi Fukuda, Kaede C. Wada, Takuro Ishii, Jun-Ichi Yonemaru, Daisuke Ogawa

**Affiliations:** 1 Research Center for Agricultural Information Technology, National Agricultural and Food Research Organization (NARO), Tokyo, Japan; 2 Institute for Agro-Environmental Sciences, NARO, Tsukuba, Japan; 3 Institute of Crop Science, NARO, Tsukuba, Japan

**Keywords:** rice, phenology, time-series analysis, MAGIC, UAV, remote sensing, transplanting protocol

## Abstract

Unmanned aerial vehicles (UAVs) are one of the most effective tools for crop monitoring in the field. Time-series RGB and multispectral data obtained with UAVs can be used for revealing changes of three-dimensional growth. We previously showed using a rice population with our regular cultivation protocol that canopy height (CH) parameters extracted from time-series RGB data are useful for predicting manually measured traits such as days to heading (DTH), culm length (CL), and aboveground dried weight (ADW). However, whether CH parameters are applicable to other rice populations and to different cultivation methods, and whether vegetation indices such as the chlorophyll index green (CIg) can function for phenotype prediction remain to be elucidated. Here we show that CH and CIg exhibit different patterns with different cultivation protocols, and each has its own character for the prediction of rice phenotypes. We analyzed CH and CIg time-series data with a modified logistic model and a double logistic model, respectively, to extract individual parameters for each. The CH parameters were useful for predicting DTH, CL, ADW and stem and leaf weight (SLW) in a newly developed rice population under both regular and delayed cultivation protocols. The CIg parameters were also effective for predicting DTH and SLW, and could also be used to predict panicle weight (PW). The predictive ability worsened when different cultivation protocols were used, but this deterioration was mitigated by a calibration procedure using data from parental cultivars. These results indicate that the prediction of DTH, CL, ADW and SLW by CH parameters is robust to differences in rice populations and cultivation protocols, and that CIg parameters are an indispensable complement to the CH parameters for the predicting PW.

## 1 Introduction

Remote sensing by unmanned aerial vehicles (UAVs) is an efficient way to phenotype crops in the field (Yang et al., 2017; Ninomiya, 2022). Typical, “multi-rotor-type” UAVs can cover a large field in a short period of time (Yang et al., 2017), and when equipped with sensing devices, they can fly over agricultural fields and acquire information about crop growth in a non-destructive manner. The sensing devices include RGB (red, green and blue) and multispectral cameras (Shi et al., 2016), and RGB images are used to reconstruct the 3D structure of plants by the structure-from-motion technique (Westoby et al., 2012). RGB and multispectral cameras are also used to quantify spectral reflectance from plants and calculate vegetation indices (VIs), which are related to yield and leaf color (Xue and Su, 2017). These data can be utilized as predictors for traits such as plant emergence (Li et al., 2019), height (Holman et al., 2016), biomass (Ogawa et al., 2021a), and yield (Wan et al., 2020). Gathering these data with manual measurements is labor-intensive for workers in the field. Therefore, UAV-based phenotyping can be more cost-effective than conventional manual methods (Reynolds et al., 2019), and is expected to facilitate agronomic studies.

Improvements of crop varieties and their cultivation methods are a necessary response to global population growth and climate change (Tester and Langridge, 2010; Heredia et al., 2022). In breeding programs, hundreds of genotypes are cultivated in the plots on a filed at the same time. Since the growing conditions suitable for cultivars vary depending on their genetic architecture (Shinada et al., 2014; Hori et al., 2016), growing tests of crops should be conducted under various cultivation conditions to account for the genetic background of the population. In terms of genetics, the methodology to analyze breeding population in multiple environments (e.g., years or locations) has been intensively studied (Crossa et al., 2022). UAV-based phenotyping is a promising technology for the usage in breeding programs (Guo et al., 2021), but its application to multi-environmental test is limited so far (e.g., Sakurai et al., 2023). A fundamental challenge is how to handle UAV-derived data for multiple genetic backgrounds, environments, or cultivation conditions. It is important for UAV-based phenotyping of crops to be robust enough to be applied to various populations under different growing conditions.

Remote sensing has enabled to trace seasonal changes in crop growth (crop phenology) using time-series observation data (Sakamoto et al., 2013; Kronenberg et al., 2021). In the case of rice, the growth process is generally divided into a vegetative growth stage before heading and a reproductive growth stage after heading. During vegetative growth, assimilates are stored in source organs (i.e., the stem and leaf), and during reproductive growth, assimilates are translocated into a sink organ (i.e., the panicle). Because the yield potential and cultivation characteristics of each cultivar or line is strongly related to the sink-source relationships (Horie et al., 1995; Li et al., 1998; Yoshida and Horie, 2009; Ohsumi et al., 2011) and the 3D architecture of plant associated with photosynthesis (Jiao et al., 2010; Khush, 2013; Burgess et al., 2017), phenology information is indispensable in agronomic studies. Many previous studies have made it possible to predict the phenology stage of rice by using VIs or image data obtained from UAVs (Desai et al., 2019; Yang et al., 2020; Ge et al., 2021; Lu et al., 2023), and various approaches to connect VI time-series data to rice phenology have also emerged (Berger et al., 2019; Yang et al., 2022). However, the number of studies that analyze genetic differences in terms of rice phenology is still limited, and the methodology to evaluate rice lines for breeding and cultivation tests by using UAV time-series data is underdeveloped.

To develop the methodology for evaluating rice lines by using UAV time-series data, two important points should be considered. First, interpretable models need to be constructed to assess which aspect of phenology the time-series data reflects. These time-series data are often used as predictors for predicting manually measured traits in machine-learning models, such as random forest, support vector regression, and neural networks, which can generally incorporate various types of UAV time-series data (Masjedi et al., 2020; Sakamoto, 2020; Shafiee et al., 2021; Xu et al., 2021). However, these complexed models are often referred to as “black box” models, meaning the difficulty in model explanation and model examination (Biecek and Burzykowski, 2021). Instead, an understanding of which predictors have what effects at which period during the crop phenology can give agronomic insight into trait prediction.

Second, appropriate methods need to be examined to deal with various cultivars and cultivation protocols. One possible approach is to divide the growth process into several developmental stages and to acquire VIs at each stage (Han et al., 2018; Wang et al., 2019; Qiu et al., 2020). However, even on the same observation date, the phenology stage of rice can vary depending on the cultivar., cultivation protocol (e.g., transplanting dates), and year because rice phenology is affected by genetic background and environment (Hori et al., 2015). Therefore, it is difficult to arrange an observation date that will target specific phenological stages of each cultivar, protocol, and year.

To tackle these two challenges, we hypothesized that applying a time-series model to explain the UAV time-series data by non-linear curve and utilizing time-series model parameters would be effective. This approach has been used for crops such as soybean (Borra-Serrano et al., 2020) and maize (Anderson et al., 2019). In our previous study (Taniguchi et al., 2022), we focused on canopy height (CH) which is the natural height of crop canopy between the ground surface and its highest point in a standing condition, and then examined its time-series trait by the UAV observations. We developed a time-series model and applied it to time-series CH data of 30 rice cultivars, including *japonica* and *indica*. The obtained model parameters (CH parameters) predicted manually measured traits such as culm length (CL) and biomass and identified relationships between the manually measured traits and model parameters. However, it is not clear whether that approach is robust enough to apply to other populations.

Moreover, in our previous study targeting 30 rice cultivars, the prediction model by CH parameters was insufficient to predict grain yield, which is a key trait in agronomic studies. According to Tsukaguchi et al. (2022), a VI related to chlorophyll content and photosynthesis activity, namely, the chlorophyll index green (CIg) (Gitelson et al., 2003; Gitelson et al., 2005), is related to rice yield over the course of growth and development. Since CIg has a sensitivity advantage compared with the Normalized Difference Vegetation Index (NDVI), a commonly used vegetation indicator, especially when the vegetation fraction tends to be saturated (Gitelson et al., 2005; Viña et al., 2011; Tsukaguchi et al., 2022), we expect it to be an appropriate VI for observing middle and late growth phases. In a study of wheat, using VIs and CH together was found to be an appropriate strategy to increase yield prediction performance (Tao et al., 2020).

This study aimed, therefore, to evaluate the performance of trait prediction models using interpretable parameters of CH and CIg as they relate to rice phenology. We developed a genetically close Multi-parent Advanced Generation Inter Cross (MAGIC) population derived from 4 *japonica* rice cultivars, which are totally different from the previous population consisting of 30 rice cultivars (Taniguchi et al., 2022), and tested the MAGIC population in paddy fields under regular and delayed transplanting protocols to investigate the influence of the different protocols on rice phenology. We extracted parameters from the CH and CIg time-series data, and quantified differences among CH and CIg parameters in terms of trait prediction. Finally, we constructed a calibration method so that trait prediction models can be applied to different transplanting protocols or different years. This study should provide valuable insights into how to obtain and handle phenological data for the prediction of manually measured traits.

## 2 Materials and methods

### 2.1 Development of Japan-MAGIC2 lines and cultivation

We used four Japanese cultivars as parents of the Japan-MAGIC2 (JAM2) lines: Iwaidawara (IW), Toyomeki (TO), Akidawara (AK), and Tachiharuka (TH). We first crossed AK with TO and IW with TH to produce seeds of two types, called the AKTO and IWTH two-way hybrids. Then these hybrids were crossed to produce four-way recombinants. We finally produced 100 JAM2 lines by the single-seed descent (SSD) method. These JAM2 lines (F5 in 2022, F6 in 2023) were used in this study (Supplementary Figure 1A).

**Figure 1:**
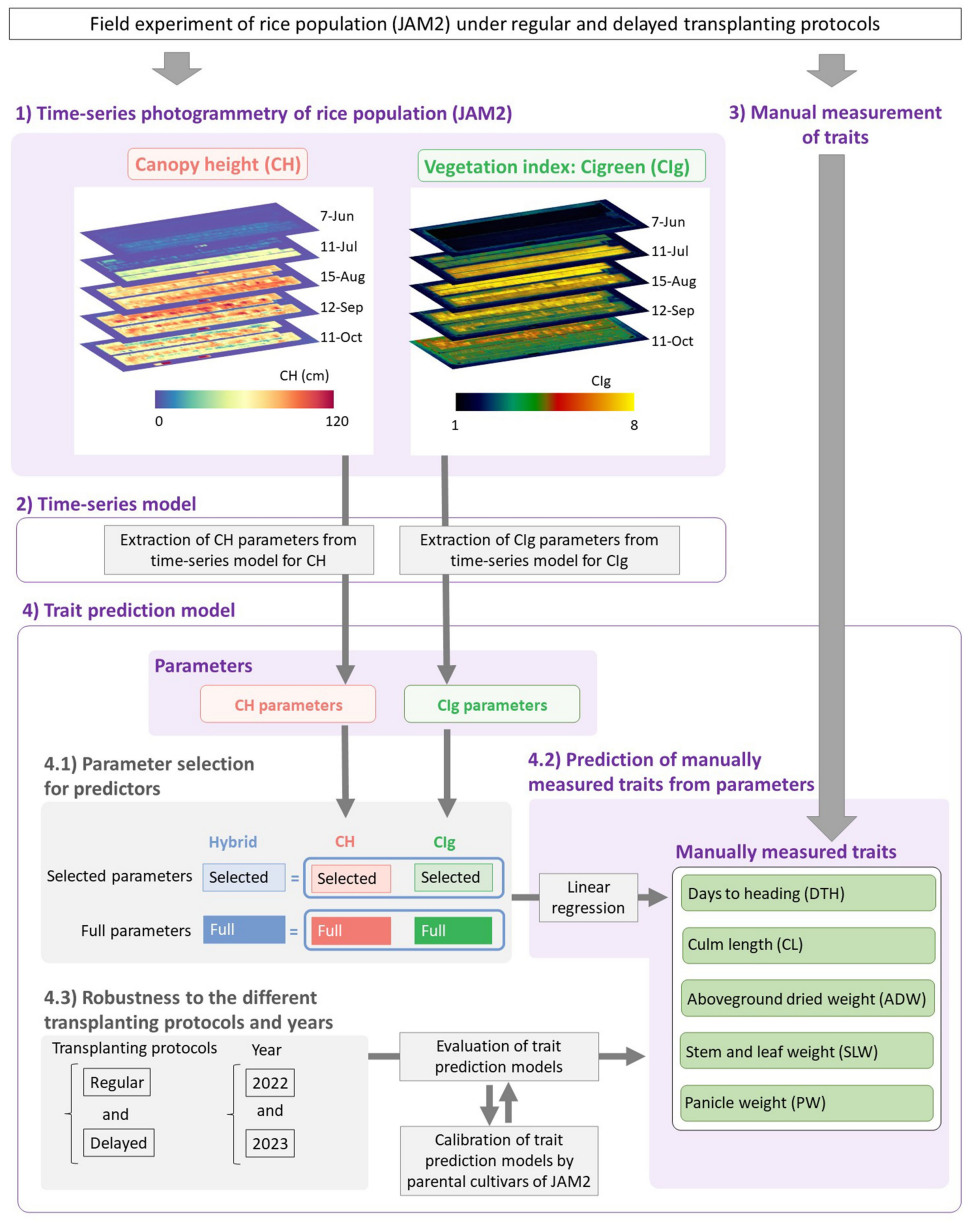
Framework of this study. (1) Time-series photogrammetry was used to extract CH and CIg data of the JAM2 lines grown in the field. (2) The CH and CIg parameters were extracted by fitting time-series models to the data. (3) Manually measured traits were obtained. (4) Linear regression models were then constructed to predict the manually measured traits from the CH and/or CIg parameters. (4.1) Six prediction models were constructed depending on the parameter types and whether variable selection was conducted or not, and model comparisons were performed. (4.2) The prediction models were applied for five manually measured traits. (4.3) The robustness of the prediction models to the different transplanting protocols and years were also evaluated.

**Figure 2:**
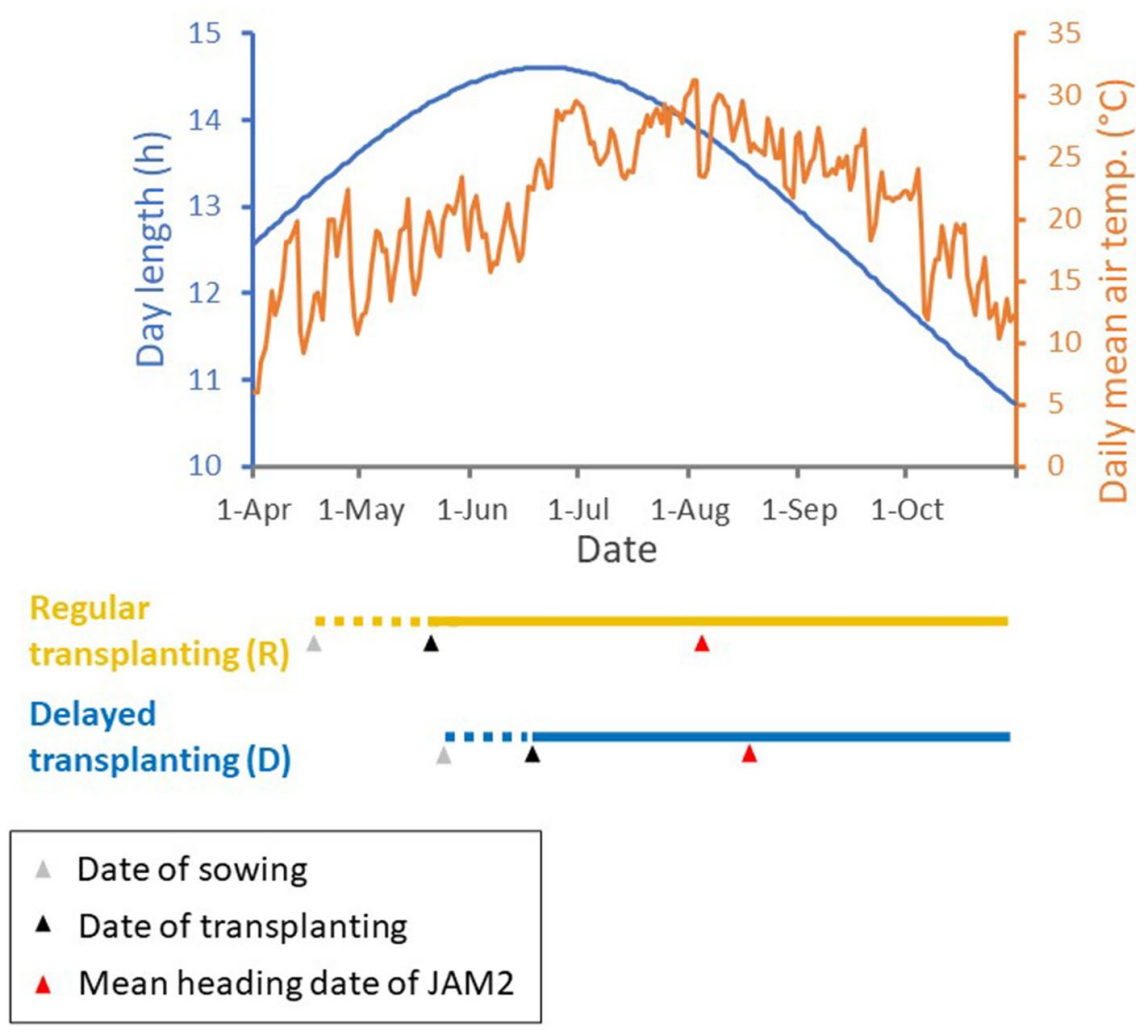
Regular and delayed transplanting protocols with meteorological data in 2022. Day length and daily mean air temperature are shown in the top graph, with the timelines of the regular and delayed transplanting protocols for the JAM2 lines shown below it. The period of rice seedling growth is shown by dotted lines, and the period of cultivation in the rice paddy field is shown by solid lines. The dates of sowing and transplanting are shown by gray and black triangles, respectively, and the mean heading dates are shown by red triangles. Monitoring of JAM2 lines with UAVs ceased at harvest. Day length and temperature data were acquired from Weather Data Acquisition System of Institute for Agro-Environmental Sciences, NARO.

We cultivated the 100 JAM2 lines in a rice field in Tsukuba, Japan, in 2022 and 2023 using both regular (R) and delayed (D) transplanting protocols (Figure 1; Supplementary Table 1). The dates of sowing and transplanting to the paddy field in the delayed transplanting protocol were about a month later than those in the regular transplanting protocol (Figure 2). In this study, the data from 2022 were used for the main analysis, and the data of 2023 were used for evaluating the robustness of the trait prediction models.

### 2.2 Manual measurements of traits related to yield

Days to heading (DTH) was scored as the number of days from transplanting rice to the field to the appearance of the first panicle in more than half of the plants in each JAM2 line. CL was assessed as the length of the longest culm of each plant measured with a ruler more than 10 days after heading. For the measurement of panicle weight (PW) and stem and leaf weight (SLW), shoots of mature plants were air-dried from one to two months in a drying room and then cut 3 cm below the panicle base to separate the parts. The aboveground dried weight (ADW) was calculated as PW + SLW. The averages of five plants in the middle lane except for the plants at both edges were used as the CL, PW, SLW, and ADW values of each JAM2 line (Figure 1).

### 2.3 UAV-based aerial photography

Similar to our previous studies (Ogawa et al., 2021b; Taniguchi et al., 2022), UAV-based aerial photography was conducted once a week to track the growth of the rice lines. For lines grown under both the regular transplanting and delayed transplanting protocols, aerial photography was conducted once a week after puddling and before the planting date until harvest, which occurred by 1 November. For the aerial photography, we used a Phantom 4 Pro (P4P) UAV equipped with an RGB camera to obtain data for calculating CH, and a DJI Phantom 4 Pro Multispectral (P4M) UAV, which is equipped with multispectral cameras, to obtain data for calculating CIg (Figure 1). The automatic flight settings for the P4M were slightly different from those for the P4P in order to shorten the flight time, thereby reducing the impact of short-term changes in incident light intensity on the spectral images (Ogawa et al., 2021a). The flight path and image shooting setting were programmed by using DJI GS Pro software. The detailed settings are in Supplementary Table 2. The band setting of P4M were as follows: blue is 450nm±16nm, green is 560nm±16nm, red is 650nm±16nm, red edge is 730nm±16nm and NIR is 840nm±16nm.

The P4P was manually raised to 10 m and the camera’s focus distance was adjusted on a region of the canopy before the aerial photography, then was started in automatic flight mode at an altitude of 10 m. The P4M was manually operated to a position 1–2 m above a Micasense Calibration Reflectance Panel (MicaSense Inc., Seattle, WA, USA) to capture spectral images of the gray plate with 50% reflectance before and after operation in automatic flight mode at an altitude of 20 m. We set painted black and white markers on paved surfaces at eight points surrounding the field as ground control points (GCPs) and then precisely measured the latitude, longitude, and altitude of each point with a TCRP1205 surveyor (Leica, Heerbrugg, Switzerland).

### 2.4 Generation of orthomosaic images and a crop surface model from the UAV images

We obtained multispectral orthomosaic images and a crop surface model (CSM) from each set of aerial images with Agisoft MetaShape Professional v. 1.7.3 software (Agisoft, St. Petersburg, Russia). The CSM was generated from the high-resolution RGB images acquired by the P4P using the same steps as described previously (Ogawa et al., 2019): (1) Align photos (accuracy, high), (2) input GCPs, (3) build dense cloud (accuracy, high), (4) build mesh (surface type, height field; source data, dense cloud), and (5) build digital elevation model (DEM; source data, dense cloud). The DEM image of 16 May was defined as the height of the ground surface. Then, a CSM image representing the height of the rice plants was created by taking the difference between the ground surface height image obtained on another observation date and the DEM image. The multispectral orthomosaic images were generated from the P4M aerial images with an alternative procedure to reduce misalignment of the spectral images (Sakamoto et al., 2022). The following steps were repeated for each spectral image: (1) Align photos (accuracy, high), (2) input GCPs, and (3) calibrate reflectance using the sun sensor data and gray panel images. The sparse-point data and the GCP data for each spectral dataset were merged into a single chunk in the Meta Shape to perform camera calibration. Then, the following steps were conducted: (4) Build DEM (source data, sparse cloud) and (5) build orthomosaic images (surface DEM; blending mode, mosaic). The orthomosaic images and the DEM images were analyzed in ENVI v. 5.5 remote sensing software (Harris Geospatial, Boulder, CO, USA). The original spatial resolution was 3 mm/pixel for P4P and 11 mm/pixel for P4M. The map projection was converted to UTM zone 54 N (WGS-84), and then both DEM and orthomosaic images were resampled with a 2-mm/pixel resolution. The resampling resolution was set to a slightly higher resolution than P4P RGB camera images because of accounting for variations in ground resolution due to changes in actual UAV flight altitude. The converted image was rotated 66° clockwise to match the direction of the long side of the field with the lateral direction of the final output image. The image was then resized to a rectangle (28,000 pixels × 14,000 pixels).

### 2.5 Quantification of CH and CIg

JAM2 lines were cultivated in a small plot in a field: the distance between adjacent plants was 30 cm between columns and 18 cm between rows. The plots in which each line was planted were cut out by using ENVI software from the orthomosaic and DEM images. Each cut-out image corresponded to 90 cm × 126 cm on the ground and contained 21 plants (Supplementary Figure 1B). From the cut-out images, CH and CIg were calculated as follows. We defined CH as the difference between the canopy position, that is, the 95th percentile value in the cut-out DSM, and the ground level; the method used to obtain values were consistent with manually measured CH data (Taniguchi et al., 2022). CIg was calculated as the ratio of NIR to green reflectance values (Gitelson et al., 2003; Gitelson et al., 2005):


$$CIg=\frac{\rho_{NIR}}{\rho_{\text{green}}}$$

We defined the CIg of each line as the mean CIg in the plot corresponding to that line in the orthomosaic image.

### 2.6 Fitting time-series model to the CH and CIg

**Figure 3:**
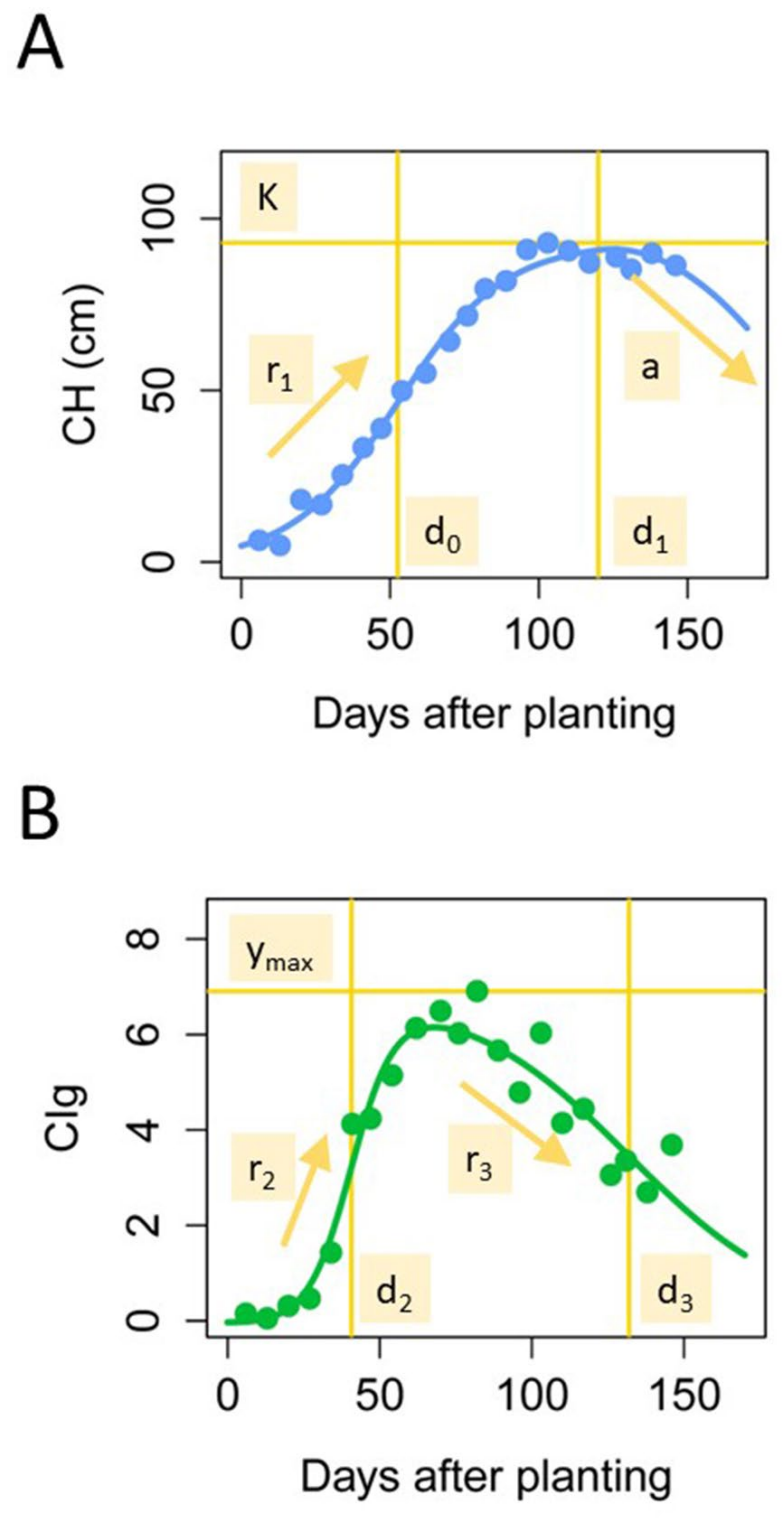
CH and CIg parameters. Trajectories of the CH **(A)** and CIg **(B)** time-series models and definitions of the model parameters.

We fitted time-series models to the CH and CIg time-series data to obtain the CH parameters and CIg parameters (Figure 1). In our previous study (Taniguchi et al., 2022), in which we analyzed time-series CH data, we applied a modified three-parameter logistic model. The three-parameter logistic model accounted for the CH decrease in the late growth period by using a quadratic curve,


$$y=\{\begin{array}{c} \frac{K}{1+\exp\left(r_{1}\left(d_{0}-x\right)\right)}\\ \frac{K}{1+\exp\left(r_{1}\left(d_{0}-x\right)\right)}-a{\left(x-d_{1}\right)}^{2} \end{array}$$

where x is the days after transplanting. The parameters of the three-parameter logistic model were estimated by the same procedure of our previous study, the algorithm of which was implemented in the R package phenolocrop (Taniguchi et al., 2022). Parameter K is the maximum value of CH, $r_{1}$ is the growth rate before the peak, $d_{0}$ is the time point at which the growth rate is a maximum, $d_{1}$ is the time point at which the maximum value of y is reached, and a is the rate at which CH decreases in the late growth period (Figure 3A). Different from CH, CIg time-series data described an S-shape with time; therefore, we adopted a double logistic model for the CIg data (Fisher and Mustard, 2007; Yang et al., 2012).


$$y=y_{\text{max}}\left(\frac{1}{1+\exp\left(r_{2}\left(d_{2}-x\right)\right)}-\frac{1}{1+\exp\left(r_{3}\left(d_{3}-x\right)\right)}\right)$$

Here, parameter $r_{2}$ is the growth rate before the peak, $r_{3}$ is the rate of decrease after the peak, $d_{2}$ is the time point at which the growth rate is a maximum, $d_{3}$ is the time point at which the decreasing rate is a maximum, and $y_{\text{max}}$ is the maximum value of CIg (Figure 3B). Here, we fitted both a three-parameter logistic model and a double logistic model to the time-series CH and CIg data. To estimate the parameters of the double logistic model, we first set the maximum value of the objective time-series variable (CH or CIg) to $y_{\text{max}}$ and then estimated the other variables in the framework of the nonlinear least squares method implemented in the R function nls. The algorithm was set to nl2sol, and the initial values of $r_{2}$, $r_{3}$, $d_{2}$, and $d_{3}$ were 0.05, 0.05, 40, and 100, respectively.

To evaluate which model was best suited to CH or CIg, we calculated the coefficients of determination ($R^{2}$) for each observation dataset. For CH, the $R^{2}$ values were high for both the three-parameter logistic model ($R^{2}=0.982$ on average) and the double logistic model ($R^{2}=0.985$ on average). While the double logistic model was slightly better, we adopted the three-parameter logistic model for the time-series CH data because our objective is to evaluate the robustness of the methodology presented in our previous study (Taniguchi et al., 2022). In contrast, for CIg, we adopted the double logistic model because the $R^{2}$ value ($R^{2}=0.941$ on average) for that model was larger than that for the three-parameter logistic model ($R^{2}=0.796$ on average; Supplementary Figure 2).

### 2.7 Characterization of traits and parameters

All of the manually measured traits and model parameters were characterized by summary statistics (mean, variance, max value, and min value), frequency distribution, and the calculated Pearson’s correlation coefficient (cor) between each trait and parameter. To determine how the CH and CIg parameters were related, we conducted principal component analysis (PCA).

### 2.8 Prediction of manually measured traits using CH and CIg parameters

For the prediction of manually measured traits from CH and/or CIg parameters, we conducted variable selection and constructed six linear regression models depending on which parameters were used as the predictors (Figure 1). The CH-selected model used *K*, *d*_0_, and *d*_1_ as predictors. These parameters were selected from the five CH parameters by a procedure called backward variable selection to prevent multicollinearity (Hastie et al., 2009). When the variance inflation factor (VIF) was calculated using the car package in R (Fox and Weisberg, 2019), the selected parameters, *K*, *d*_0_, and *d*_1_, had VIFs lower than 5 (Supplementary Table 3). The CIg-selected model used $r_{2}$, $d_{2}$, $d_{3}$, and $y_{\text{max}}$ as predictors without multicollinearity. These parameters were selected from the five CIg parameters in the same way (Supplementary Table 4). Then, to investigate whether the two models contained different information for trait prediction, we constructed a “Hybrid-selected model,” which used all of the predictors used by the CH-selected and CIg-selected models. We also constructed the corresponding full models (CH-full model, CIg-full model, and Hybrid-full model) to examine the effects of variable selection.

**Figure 4:**
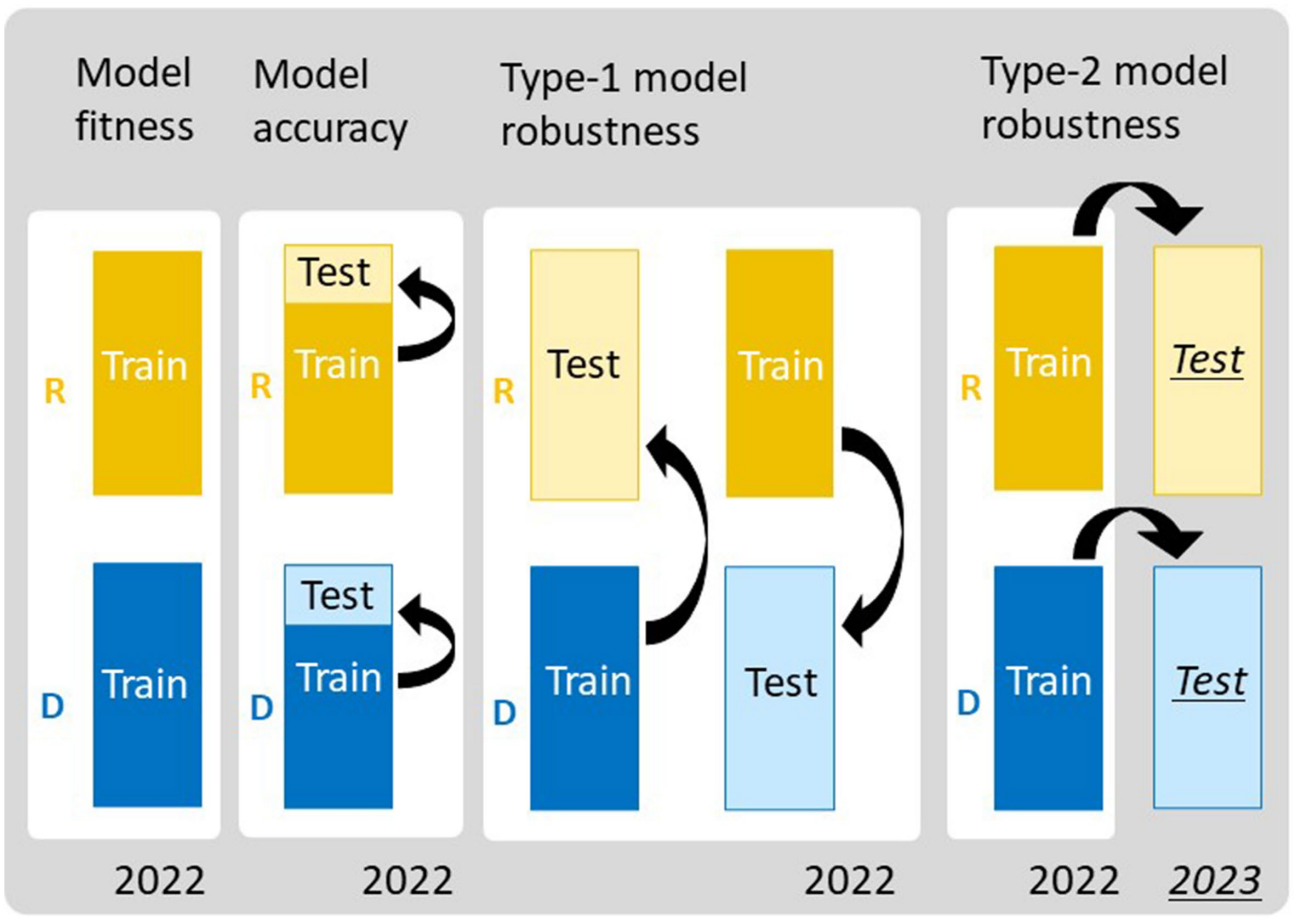
Frameworks for evaluating the performance of the prediction models. To assess model fitness, all data of regular (R) and delayed (D) transplanting protocols were treated as training data. To assess model accuracy, 10-fold cross-validation (10-CV) was used, in which each dataset was split into training and test data. Type-1 model robustness was assessed by using data from different transplanting protocols as training and test data. Type-2 model robustness was assessed by using the data from 2023 as the test data and those from 2022 as the training data.

We evaluated the performance of the six prediction models using four validation schemes: model fitness, model accuracy, type-1 model robustness, and type-2 model robustness (Figure 4). To evaluate model fitness, each prediction model was fitted separately to the JAM2 data of R and D in 2022, and their coefficients of determination ($R^{2}$), regression coefficients, and *p*-values were calculated to measure the goodness of fit and to identify those parameters that were important for trait prediction. To evaluate model accuracy, we conducted 10-fold cross validation (10-CV) for R and D separately in 2022. Because cross-validation results can fluctuate depending on the data-splitting process, we randomly repeated the 10-CV 100 times and calculated root mean squared errors (RMSEs) and cor values. We also compared prediction accuracies among the CH-selected, CIg-selected, and Hybrid-selected models by conducting a “Tukey-like” non-parametric multiple comparison among them with the nparcomp function implemented in the nparcomp package in R (Konietschke et al., 2015). To evaluate type-1 model robustness, we trained the prediction model with R data and tested the trained model against D data, and vice versa. To evaluate type-2 model robustness, we trained the prediction model with R data of 2022 and tested the trained model against R data of 2023. We also trained the prediction model with D data of 2022 and tested the trained model against D data of 2023. We quantified the two types of model robustness by calculating the RMSE and the cor values.

### 2.9 Calibration of the prediction models for improving the model robustness

After predicting the phenotypic values of the test data in the evaluation procedure of model robustness, we calibrated the predicted phenotypic values by using the data of the parental cultivars of JAM2. The calibration model was constructed in the following five steps (Supplementary Figure 3).

As shown in Supplementary Figure 3A, for the type-1 model robustness, (1) we trained the prediction model with D data of JAM2. (2) We applied the trained prediction model to R data of JAM2 and obtained the predicted values of R data of JAM2. (3) We applied the trained prediction model to R data of parental cultivars of JAM2 and obtained the predicted values of the parental cultivars. (4) We trained a single regression model as the calibration model by comparing the predicted and observed values of the parental cultivars. (5) We applied the trained calibration model to the predicted values of the R data of JAM2 and obtained the calibrated values. Similarly, for the case of training the prediction model with R data of JAM2, we calibrated the predicted values of D data of JAM2. We quantified the calibration by calculating the RMSE and the cor values.

For the type-2 model robustness, as shown in Supplementary Figure 3B, (1) we trained the prediction model with R data of JAM2 in 2022. (2) We applied the trained prediction model to R data of JAM2 in 2023 and obtained the predicted values of R data of JAM2 in 2023. (3) We applied the trained prediction model to R data of the parental cultivars of JAM2 in 2023 and obtained the predicted values of the parental cultivars. (4) We trained a single regression model as the calibration model by comparing the predicted and observed values of the parental cultivars. (5) We applied the trained calibration model to the predicted values of R data of JAM2 in 2023 and obtained the calibrated values. Similarly, for the case of training the prediction model with D data of JAM2 in 2022, we calibrated the predicted values of D data of JAM2 in 2023. These calibration frameworks were applied to the Hybrid-selected model; the parameter and trait data are available in Supplementary Data 1.

## 3 Results

### 3.1 Differences in manually measured traits in the JAM2 lines between regular and delayed transplanting protocols

**Figure 5:**
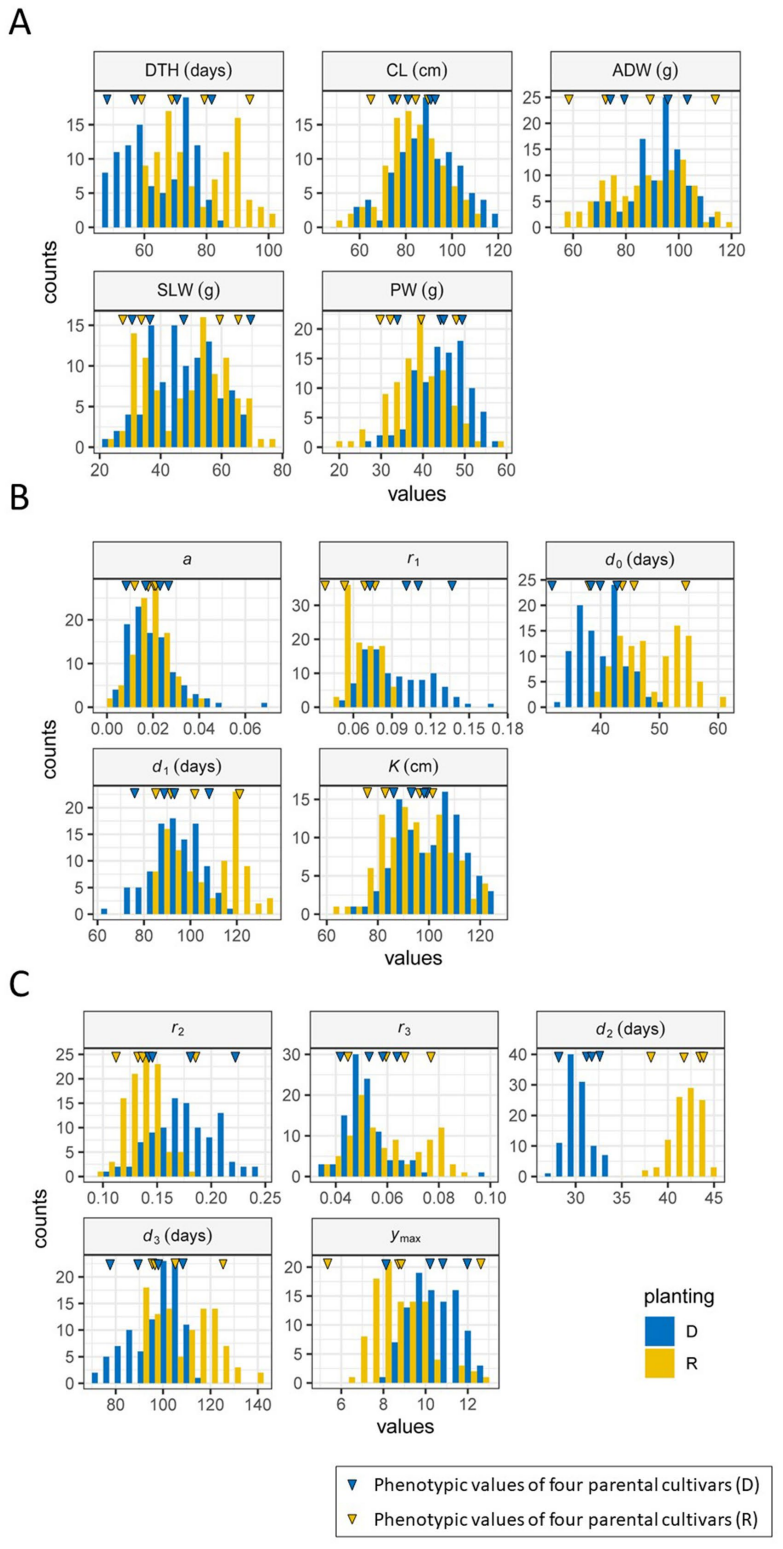
Frequency distributions of traits and parameters in 2022. **(A)** Manually measured traits. **(B)** CH parameters. **(C)** CIg parameters. R (yellow): regular transplanting, and D (blue): delayed transplanting. The blue (D) and yellow (R) inverted triangles indicate the phenotypic values of the four parental cultivars. DTH, days to heading; CL, culm length; ADW, aboveground dried weight; SLW stem and leaf weight; PW, panicle weight.

We examined the distributions of five manually measured traits of the JAM2 lines grown under the regular and delayed transplanting protocols. The ranges of trait distribution under the regular transplanting protocol were as follows: days to heading (DTH: 59–100 days), culm length (CL: 51–110 cm), aboveground dried weight (ADW: 56–118 g), stem and leaf weight (SLW: 24–76 g), and panicle weight (PW: 21–58 g). Those under the delayed transplanting protocol were as follows: DTH (48–84 days), CL (58–121 cm), ADW (69–114 g), SLW (23–70 g), and PW (28–59 g). We found that the CL, ADW, SLW, and PW distributions, which were investigated at the maturation stage, were largely comparable between the regular and the delayed transplanting protocols. By contrast, the DTH distribution was clearly shorter under the delayed transplanting protocol (48–84 days) than under the regular transplanting protocol (59–100 days). Thus, the vegetative growth period was shortened under the delayed transplanting protocol (Figure 5A; Supplementary Table 5).

Positive correlations were detected between the regular and delayed transplanting protocols for the five manually measured traits, with correlation coefficients ranging from 0.98 (DTH) to 0.50 (PW: Supplementary Figure 4). In the case of DTH, which had the highest correlation coefficient, the transition from the vegetative phase to the reproductive phase was consistently earlier under the delayed transplanting protocol. The probable reason is that the JAM2 parental cultivars are adapted to the Japanese environment, so the transition occurs when the plants detect high temperatures together with a shorter day length at the end of July (Figure 2), conditions that are known to promote heading (Vicentini et al., 2023). The correlation coefficient of CL (0.96) was also high, but the difference between the transplanting protocols in this trait was small. The correlation for PW was small (cor = 0.50) but still significantly positive (Supplementary Table 4). The correlations for ADW and SLW were between these values at 0.65 and 0.81, respectively.

Among the five traits, DTH was consistently highly correlated to SLW (cor > 0.7; Supplementary Figure 5). The correlation of CL with SLW was smaller but still positive (about 0.6). Under the regular transplantation protocol, PW showed almost no correlation with DTH or SLW, whereas under the delayed transplanting protocol, PW was negatively correlated with DTH and SLW. Thus, PW exhibited distinctive characteristics under the delayed protocol.

Overall, these results indicate that these five traits have distinctive characteristics in terms of influence by different transplanting protocols and correlations among traits. It is therefore reasonable to focus on the growth pattern of the JAM2 lines during cultivation to predict the five manually measured traits.

### 3.2 Characteristics of CH and CIg parameters in JAM2 lines

We first focused on the vertical growth pattern through time-series CH monitoring. From temporal CH data of the JAM2 lines, we extracted the CH parameters from the time-series image data from the UAVs, which reflect the vertical growth of rice and consist of a, $d_{0}$, $d_{1}$, $r_{1}$, and K (Figure 3A). The frequency distributions of the growth speed $r_{1}$, the time point of maximum growth speed $d_{0}$, and the time point of maximum CH $d_{1}$ in the modified three-parameter logistic model of time-series CH data differed notably between the two protocols (Figure 5B).

Next, we focused on CIg, an index of the total chlorophyll content of the canopy that reflects vegetation amount and senescence. The pattern of the CIg curve was similar to that of the CH curve, but the maximum CIg was reached at an earlier date than the CH maximum (Figures 3A,B). We fitted a double logistic model to the time-series CIg data of the JAM2 lines to determine the five CIg parameters $r_{2}$, $r_{3}$, $d_{2}$, $d_{3}$, and $y_{\text{max}}$. The frequency distributions of all CIg parameters differed between the regular and delayed transplanting protocols, but the distributions of growth rate $r_{2}$ and the time point of the maximum growth rate $d_{2}$ differed more remarkably between the protocols than those of the other CIg parameters (Figure 5C). These results indicates that early growth might be sensitive to the cultivation protocol.

**Figure 6:**
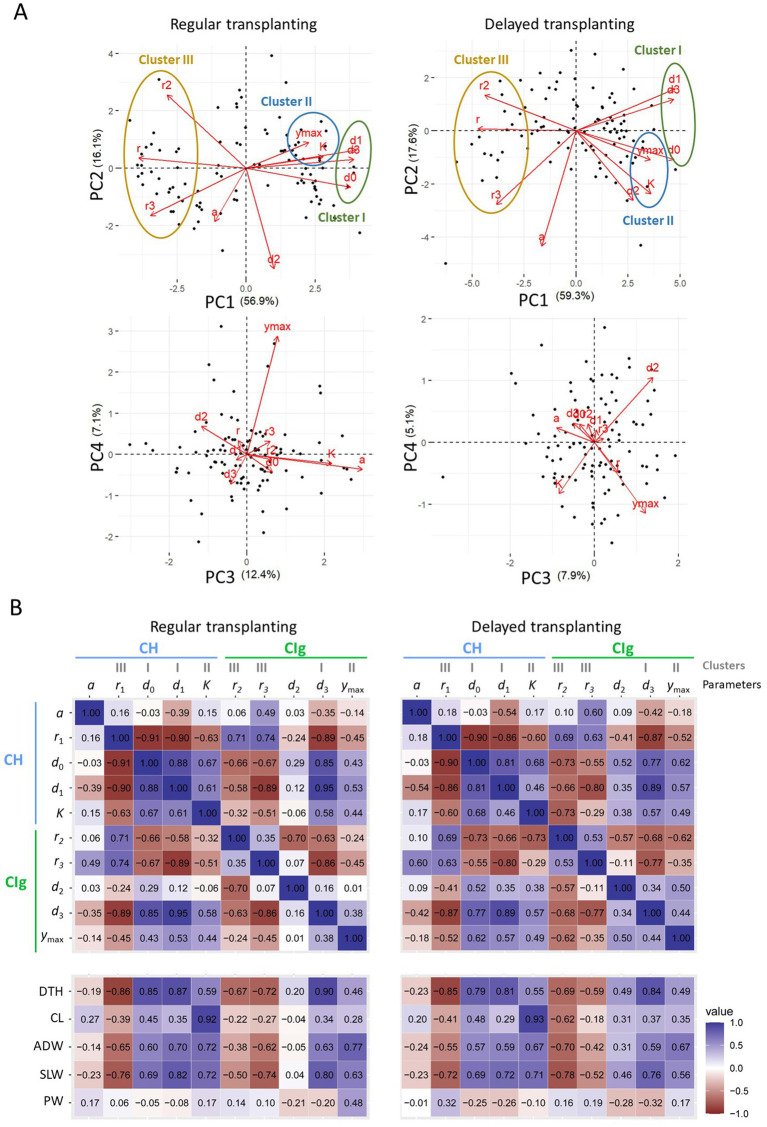
Relations between CH and CIg parameters and between the parameters and measured traits in 2022. **(A)** PCA results for CH and CIg parameters of data obtained under regular (left panel) and delayed (right panel) transplanting protocols. (upper) Biplots of PC1 and PC2; (lower) biplots of PC3 and PC4. The proportion of the total variance contributed by each principal component is shown in parenthesis. Black points indicate data of JAM2 lines, and red arrows indicate the correspondence of CH and CIg parameters to the PCA space. **(B)** Correlation coefficients between parameters for the regular transplanting (left) and delayed transplanting (right) protocols. The parameters are defined in 2.6. DTH, days to heading; CL, culm length; ADW, aboveground dried weight; SLW stem and leaf weight; PW, panicle weight.

Focusing on PC1 in the PCA (Figure 6A), we found three parameter clusters common to both the regular and delayed transplanting protocols: cluster I (consisting of $d_{0}$, $d_{1}$, and $d_{3}$), cluster II (K and $y_{\text{max}}$), and cluster III ($r_{1}$, $r_{2}$, and $r_{3}$). The parameters in each cluster were positively correlated (Figure 6B). Notably, the parameters of clusters I and III, which reflect key time points and rates of growth and development, respectively, were negatively correlated. Parameter $d_{2}$ was also negatively correlated with $r_{2}$. These results reflect the tendency for faster growth to be associated with a shorter period of growth and development and vice versa. The reproductive phase parameters a and $r_{3}$ were weakly correlated. In addition, K and $y_{\text{max}}$, both in cluster II, were weakly correlated but contained different information, as can be seen by examining PC3 and PC4 (Figure 6A). Considered together, these results show that the CH and CIg parameters had both similar and distinct characteristics.

### 3.3 Fitness of the prediction models using CH and CIg parameters

**Figure 7:**
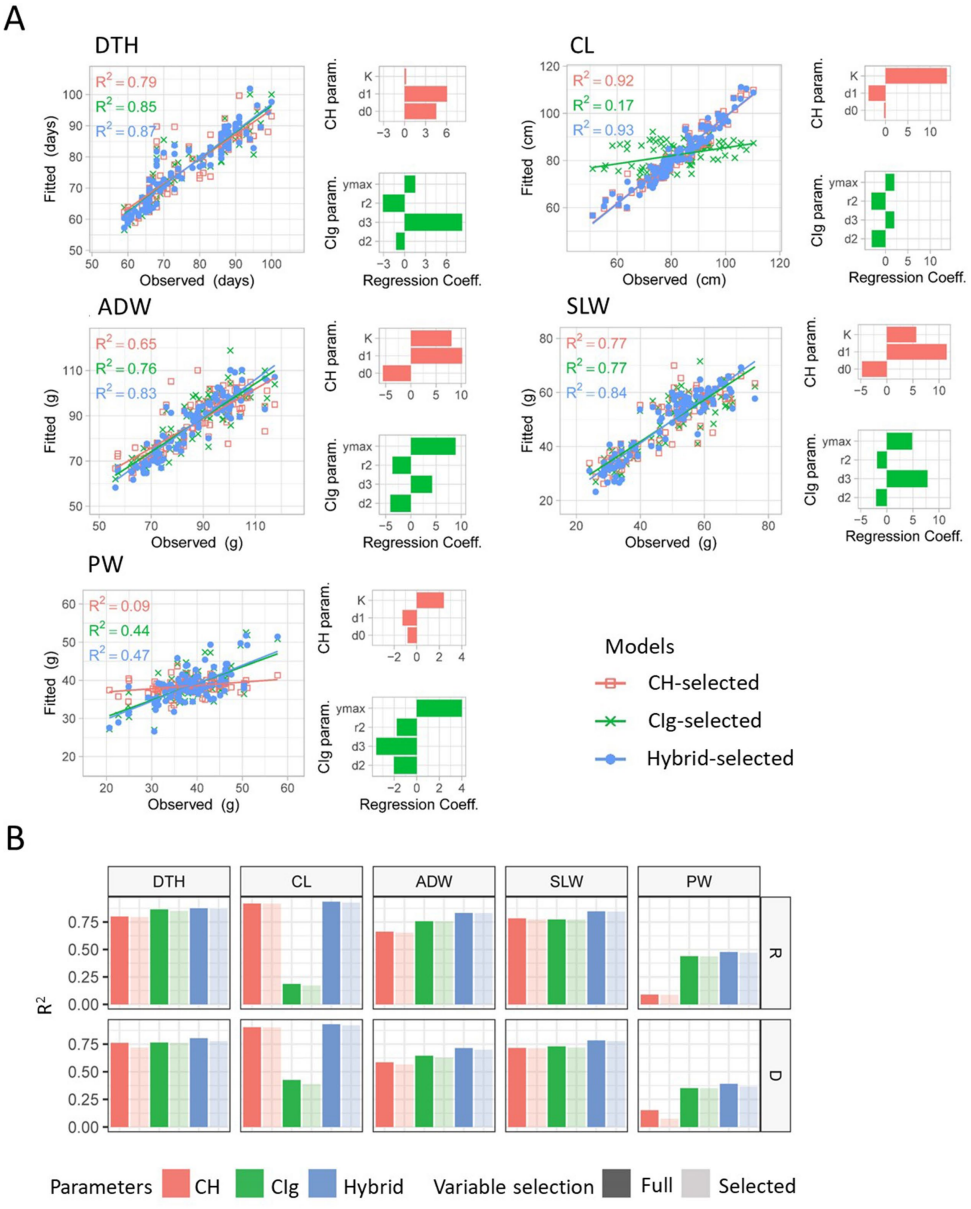
Model fitness for prediction of the dependent variables CL, DTH, ADW, SLW, and PW. **(A)** Model fitness results when regression models with the selected predictors were fitted to the regular transplanting protocol data. Scatter plots show the relations between the fitted values and the observed values. The CH-, CIg-, and Hybrid-selected models are distinguished by color. *R*^2^ values shown in each scatter plot represent coefficients of determination between fitted and observed values. Bar plots show the regression coefficients of each selected independent variable of the CH or CIg model. **(B)** *R*^2^ values for each dependent variable when regression models were fitted to the regular (R) or delayed (D) transplanting data. The colors indicate which predictors were used (CH, CIg, or Hybrid); light colors show the results when selected parameters were used, and dark colors show the results when full parameters were used. DTH, days to heading; CL, culm length; ADW, aboveground dried weight; SLW stem and leaf weight; PW, panicle weight.

We evaluated the model fitness based on the goodness of fit of the prediction models to the training data by considering $R^{2}$ (Figures 4, 7). Furthermore, we examined which CH parameters had large effects on model fitness. With regard to the prediction of DTH, $R^{2}$ was between 0.7 and 0.8 for both protocols (Supplementary Table 6). The coefficient of $d_{1}$ on DTH had the largest absolute value and was significantly positive (Figure 7A; Supplementary Tables 7, 8). For the prediction of CL, $R^{2}$ was more than 0.9 under both protocols. The coefficient of K on CL had the largest absolute value and was significantly positive. For the prediction of SLW and ADW, $R^{2}$ was between 0.5 and 0.8 under both protocols. The coefficient of $d_{1}$ on SLW had the largest absolute value and was significantly positive. For the prediction of ADW, the absolute value of the coefficient of $d_{1}$ was largest under the regular transplanting protocol, whereas the effect of K was largest under the delayed transplanting protocol. For the prediction of PW, $R^{2}$ was close to zero for all CH parameters (Figure 7B; Supplementary Table 6). These results indicate that the model using CH parameters was fit to predict DTH, CL, ADW, and SLW but not PW. More specifically, $d_{1}$ and K contained rich information for the prediction model to fit the data of DTH, CL, ADW, and SLW in the JAM2 lines, but there were no CH parameters to fit the data of PW.

We also asked if CIg would be useful for the prediction models to fit to the training data of manually measured traits. Since CIg had a higher correlation to PW than NDVI, especially during the early growth period (Supplementary Figure 6), we adopted CIg as the focal VI. With regard to the prediction of DTH, $R^{2}$ was consistently greater than 0.7 and the absolute value of the coefficient of $d_{3}$ was largest and significantly positive (Figure 7A; Supplementary Tables 6, 9, 10). For the prediction of CL, $R^{2}$ was 0.17 for the regular transplanting protocol and 0.39 for the delayed transplanting protocol; therefore, CIg parameters were not appropriate for predicting CL. For the prediction of ADW and SLW, $R^{2}$ was between 0.6 and 0.8 for both protocols, and the absolute value of the coefficient of $y_{\text{max}}$ was largest for the regular transplanting protocol, whereas that of $r_{2}$ was largest for the delayed transplanting protocol. For the prediction of SLW, the coefficient of $d_{3}$ consistently had the largest absolute value and was significantly positive. For the prediction of PW, $R^{2}$ was 0.44 for the regular transplanting protocol and 0.35 for the delayed transplanting protocol. The coefficient of $y_{\text{max}}$ had the largest absolute value and was significantly positive. The coefficient of $d_{3}$ on PW had the second largest absolute value and was significantly negative. These results indicate that the model using CIg parameters was fit to predict DTH, ADW, SLW, and PW but not CL. Specifically, $r_{2}$, $y_{\text{max}}$, and $d_{3}$ in the CIg parameters contained information for the prediction model to fit the data of DTH, ADW, SLW, and PW in the JAM2 lines, but there were no CIg parameters to fit the data of CL.

To improve predictions of the five manually measured traits, we attempted to use both CH and CIg parameters for prediction (Hybrid-selected model). For all five traits obtained under regular and delayed transplanting protocols, the $R^{2}$ of the Hybrid-selected model was higher than that of either the CH-selected or the CIg-selected model (Figure 7B; Supplementary Table 6). This result indicates that the use of CH and CIg parameters together increases model fitness. We also compared CH-selected, CIg-selected, and Hybrid-selected models with CH-full, CIg-full, and Hybrid-full models. The effects of variable selection on model fitness are described in the Discussion.

### 3.4 Accuracy and robustness of the prediction models using CH and CIg parameters

**Figure 8:**
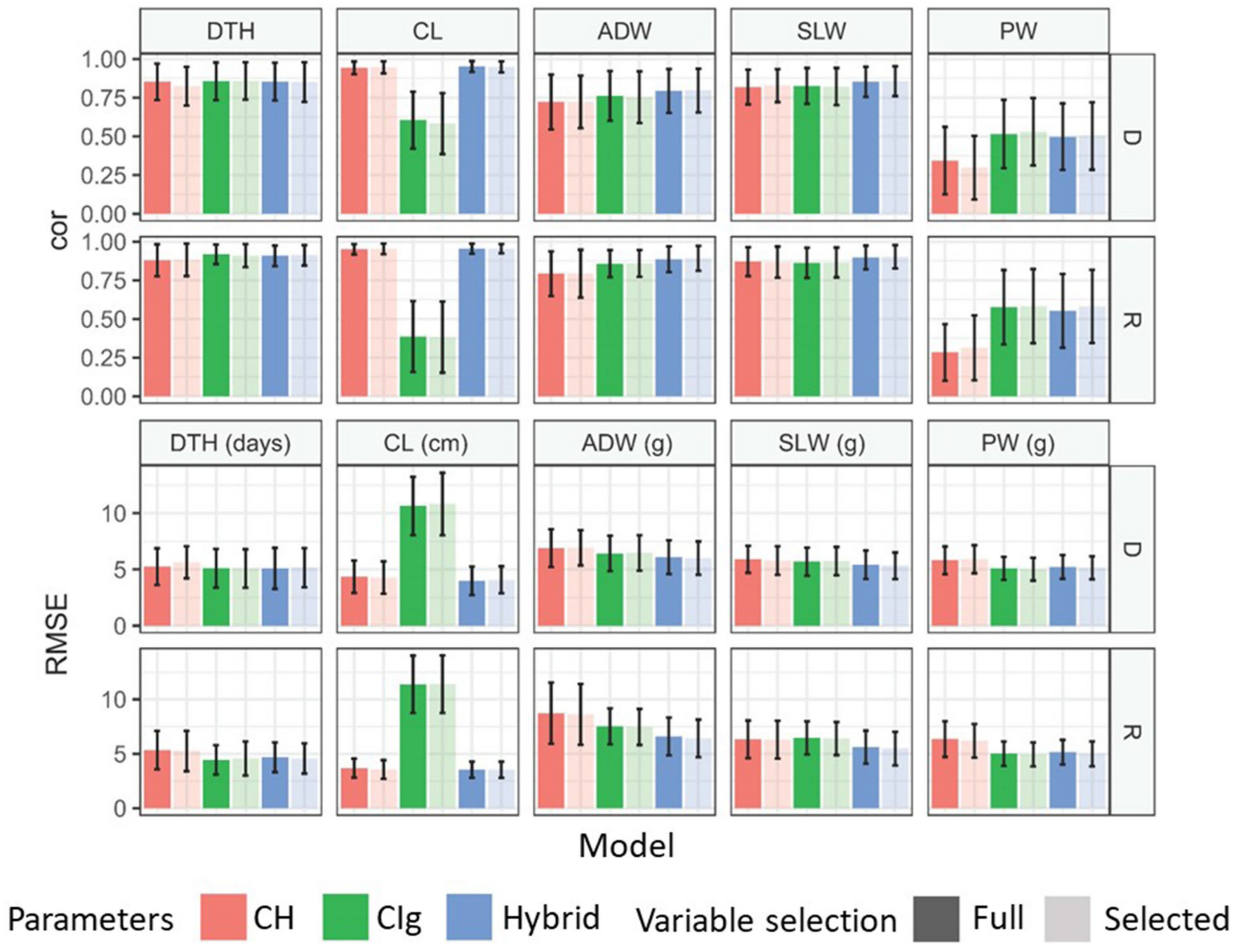
Model comparison for the prediction of each trait under model accuracy. Mean correlations and RMSEs obtained by 100 repetitions of 10-CV. Error bars indicate the standard deviation. Models were separately applied to delayed (D) and regular (R) transplanting protocol data. See Figure 5 for trait abbreviations. DTH, days to heading; CL, culm length; ADW, aboveground dried weight; SLW stem and leaf weight; PW, panicle weight.

Model accuracy was evaluated based on the ability to predict test data obtained under the same conditions (transplanting protocol and year) as the training data (Figure 4). The model accuracy of each trait by the CH-selected, CIg-selected, and Hybrid-selected models was consistent with the model fitness both in terms of cor and RMSE between predicted and observed values (Figure 8; Supplementary Figure 7 and Supplementary Table 11). For the prediction of PW, the CIg-selected model showed significantly better performance than CH-selected model (Supplementary Figure 7).

**Figure 9:**
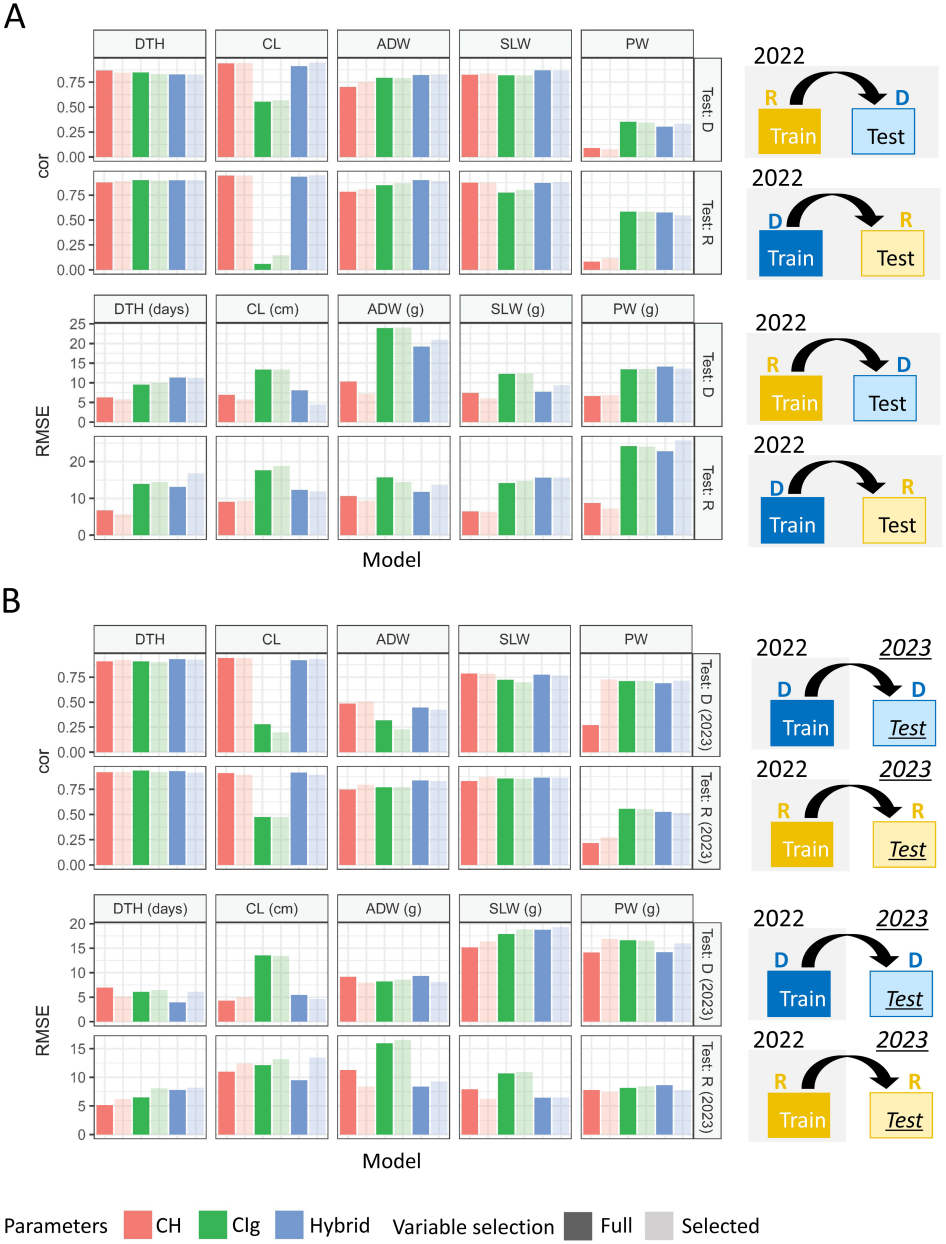
Model comparison for the prediction of each trait under model robustness. Correlation coefficients and RMSEs obtained by applying the prediction models to test data. **(A)** Under type-1 model robustness, cor values and RMSEs of each protocol were plotted for 2022: tested against data of delayed transplanting protocol (Test: D) or tested against data of regular transplanting protocol (Test: R). **(B)** Under type-2 model robustness, cor values and RMSEs of each protocol were plotted: Test: D (2023) or Test: R (2023). DTH, days to heading; CL, culm length; ADW, aboveground dried weight; SLW stem and leaf weight; PW, panicle weight.

Model robustness was evaluated based on the ability to predict test data obtained under different transplanting protocols (type-1) or different years (type-2) from the training data (Figure 4). For DTH, CL, and SLW, the correlations between predicted and observed values in type-1 and -2 model robustness were consistent, but RMSEs tended to fluctuate and become larger (Figure 9). For example, when DTH was used to evaluate type-1 model robustness, CH-selected, CIg-selected, and Hybrid-selected models had similarly high cor values, whereas the RMSEs were different; CIg-selected and Hybrid-selected models had larger RMSEs than the CH-selected model (Supplementary Table 12), possibly because the values predicted by the former two models are biased to larger values (Supplementary Figure 8).

In the case of the prediction of ADW using CH-selected, CIg-selected, and Hybrid-selected models, cor values were high in terms of type-1 model robustness but not in terms of type-2 model robustness (Supplementary Tables 12, 13). For the prediction of PW by CIg-selected and Hybrid-selected models, which had high model fitness and accuracy, cor values were not always high (Supplementary Table 13). A bias in the predicted values of ADW and PW was also observed (Supplementary Figure 8).

In summary, except for the prediction of PW and ADW in some cases, the prediction models with high model accuracy also had high model robustness in terms of cor, but they also tended to have larger RMSEs because of prediction biases derived from different transplanting protocols and years. The effect of variable selection is considered in the Discussion.

### 3.5 Calibration of training and test data obtained under different protocols

**Figure 10:**
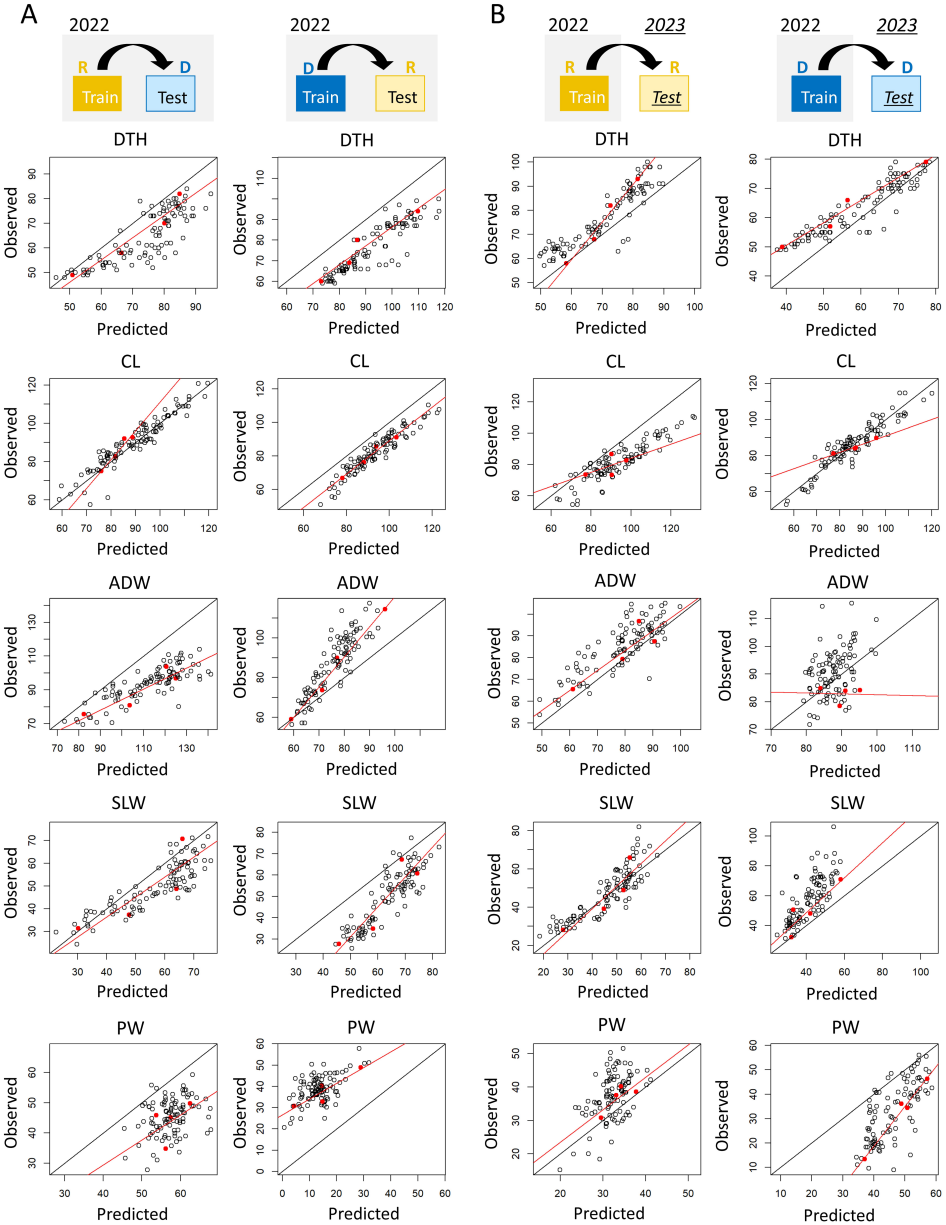
Visualization of the calibration process. Each panel shows the predicted and observed values of each manually measured trait in terms of **(A)** type-1 model robustness and **(B)** type-2 model robustness. Black points are JAM2 lines, and red points are the parental cultivars used for calibration. The red lines represent calibration models trained by using the parental cultivars, and the black lines represent a one-to-one correspondence between predicted and observed values. DTH, days to heading; CL, culm length; ADW, aboveground dried weight; SLW stem and leaf weight; PW, panicle weight. R, regular transplanting protocol; D, delayed transplanting protocol.

To mitigate the problem of large RMSEs when the training and test data were from different transplanting protocols or years, we evaluated the improvement by a calibration procedure using training and test data (Figure 10). In terms of type-1 model robustness, the calibration reduced the RMSE in predicting DTH by the Hybrid-selected model from 11.3 to 6.81 (trained using R data) and from 16.7 to 5.90 (trained using D data; Supplementary Table 14). The calibration also reduced the RMSE values for the prediction of ADW, SLW, and PW (Supplementary Table 14). There was one case where calibration resulted in a larger RMSE: the prediction of CL under the delayed transplanting protocol. In terms of type-2 model robustness, calibration reduced the RMSE values for PW (Supplementary Table 15), but the calibration did not always work well for CL, ADW, DTH and SLW. In these cases, the phenotypic data of the four parental cultivars did not cover the full range of phenotypic variance of the JAM2 lines.

## 4 Discussion

One of the most important challenges in time-series phenotyping is the extraction of essential information from individual time period image data, which can be one of the features of a crop line under a given environment. We previously found that five CH parameters derived from time-series CH data are useful for analyzing the process of growth and development of 30 genetically diverse rice cultivars during an experiment lasting 3 years and, furthermore, for predicting manually measured traits such as DTH, CL, ADW, and SLW (Taniguchi et al., 2022). In this study, we extracted the CH parameters from different JAM2 rice lines grown under both regular and delayed transplanting protocols. We confirmed that the CH parameters were useful for predicting DTH, SLW, ADW, and CL, but not PW. We defined five CIg parameters from time-series VI data for the first time. By analyzing the characteristics of CH and CIg parameters, we found that the CH and CIg parameters had both similar and distinct characteristics. CIg parameters could predict the yield trait PW in addition to DTH, ADW, and SLW, but not CL. Use of both CH and CIg parameters enabled the prediction of all of the manually measured traits we focused on in this study. These results show that time-series monitoring of both vertical and lateral growth using UAVs in the field can potentially substitute for laborious and time-consuming manual phenotyping.

In this study, $d_{0}$, $d_{1}$, and K were selected from the five CH parameters as variables for trait prediction in JAM2 lines. These CH parameters were also selected for trait prediction in 30 rice cultivars by Taniguchi et al. (2022). The parameters that contributed the most to the prediction of DTH, CL, and SLW were consistent between this study and the previous study (Taniguchi et al., 2022). For the prediction of ADW, the effects of $d_{1}$ were large and positive in this and previous studies (Taniguchi et al., 2022). These results indicate the versality of CH parameters in trait prediction.

To describe the serial dynamics of CIg with a limited number of parameters, we introduced a double logistic model. Double logistic models have previously been used for the extraction of plant phenology of forests (Fisher and Mustard, 2007; Yang et al., 2012) and agricultural crops (Liu and Zhan, 2016; Son et al., 2016; Guo et al., 2022). In this study, goodness of fit of the double logistic model to CIg time-series data was very high, indicating that a small number of CIg parameters could describe much of the phenological dynamics of CIg. By comparing CIg parameters from different transplanting protocols, we succeeded in identifying growth patterns differences, which are also analyzed by CH parameters.

For the prediction of PW, CIg parameters showed a sufficiently high level of performance in terms of model fitness and model accuracy. Although the parameter with the largest effect was $y_{\text{max}}$, it was not strongly related to the CH parameters, which suggests that $y_{\text{max}}$ contains some important information about PW that is not contained in the CH parameters. According to Tsukaguchi et al. (2022), the number of rice spikelets can be explained by CIg, especially 15 days before heading. They suggested that this result is consistent with previous studies showing the importance of nitrogen accumulation during the late spikelet differentiation stage (Yoshida et al., 2006; Kamiji et al., 2011). Parameter $y_{\text{max}}$ may correspond to the CIg 15 days before heading. Because spikelet number is related to yield (Sheehy et al., 2001), it is reasonable to assume that CIg contributes to PW as well. The parameter $d_{3}$ had the next largest effect on PW, and its effect was negative, whereas the effect of $d_{3}$ on SLW was positive. Considering that a higher $d_{3}$ means a longer term with high vegetation, these results may reflect on the trade-off between PW and SLW during maturation in the reproductive phase.

Generally, one of the goals of variable selection is to avoid multicollinearity, which causes problems in calculating regression coefficients and in interpreting prediction models. However, variable selection can decrease model flexibility, thereby losing information to fit to the training data. To assess the influence of variable selection, we compared CH-selected, CIg-selected, and Hybrid-selected models to their corresponding full models (CH-full, CIg-full, and Hybrid-full models). The difference in $R^{2}$ values between the full models and selected models was very small (0.012 on average), indicating that the information loss was negligible (Supplementary Table 6). Therefore, in this case, variable selection was a useful procedure that enabled the detection of important CH and CIg parameters for trait prediction by precisely calculating regression coefficients without information loss.

In this study, we found that the prediction models using CH and/or CIg parameters (CH-selected, CIg-selected, and Hybrid-selected models) performed consistently well when the training and test data were from the same transplanting protocol and year. When the transplanting protocols and years were different, however, RMSEs increased and fluctuated, depending on the prediction models (Figure 9; Supplementary Tables 12, 13). Similar problems were also observed when comparing the selected models to the corresponding full models. These results indicate that the test data contained some additional factors, derived from the difference in transplanting protocols or years, which acted as random noise causing bias in predicted values. To reduce the bias associated with differences of transplanting protocols or years, we developed a calibration procedure using parental cultivars and confirmed its effectiveness. This was achieved by developing a calibration model that could numerically measure and correct the bias in the predicted values. The calibration did not work well for some cases. This may be because the phenotypic data of the four parental cultivars did not cover the full range of phenotypic variance of the JAM2 lines. These results indicate that use of appropriate actual values for the calibration is effective for prediction using training data obtained under different environments.

Here, we presented a methodology for the prediction of manually measured traits from time-series image data via CH and CIg parameters. These parameters were useful for comparisons among crop lines in terms of phenology. So far, we have developed a haplotype-based genome-wide association study method using the MAGIC rice population (Ogawa et al., 2018a; Ogawa et al., 2018b), and we have revealed quantitative trait loci for the vegetation fraction and CH from time-series data in the field (Ogawa et al., 2021a,b). By combining UAV-based time-series phenotyping data and genomic information, it should be possible to analyze the phenotypic values in terms of phenology, genetics, and transplanting protocols in more detail. It remains a future challenge to develop a more comprehensive method that combines the use of genomic and UAV information for additional improvements of trait prediction.

## Data Availability

The original contributions presented in the study are included in the article/Supplementary material, further inquiries can be directed to the corresponding authors.

## References

[ref1] Anderson S. L. Murray S. C. Malambo L. Ratcliff C. Popescu S. Cope D. . (2019). Prediction of maize grain yield before maturity using improved temporal height estimates of unmanned aerial systems. Plant Phenome J. 2, 1–15. doi: 10.2135/tppj2019.02.0004

[ref3] Berger A. Ettlin G. Quincke C. Rodríguez-Bocca P. (2019). Predicting the normalized difference vegetation index (NDVI) by training a crop growth model with historical data. Comput. Electron. Agric. 161, 305–311. doi: 10.1016/j.compag.2018.04.028

[ref4] Biecek P. Burzykowski T. (2021). Explanatory model analysis. New York: Chapman and Hall/CRC.

[ref5] Borra-Serrano I. De Swaef T. Quataert P. Aper J. Saleem A. Saeys W. . (2020). Closing the phenotyping gap: high resolution UAV time series for soybean growth analysis provides objective data from field trials. Remote Sens. 12:1644. doi: 10.3390/rs12101644

[ref6] Burgess A. J. Retkute R. Herman T. Murchie E. H. (2017). Exploring relationships between canopy architecture, light distribution, and photosynthesis in contrasting Rice genotypes using 3D canopy reconstruction. Front. Plant Sci. 8:734. doi: 10.3389/fpls.2017.00734, PMID: 28567045 PMC5434157

[ref7] Crossa J. Montesinos-López O. A. Pérez-Rodríguez P. Costa-Neto G. Fritsche-Neto R. Ortiz R. . (2022). “Genome and environment based prediction models and methods of complex traits incorporating genotype × environment interaction” in Genomic prediction of complex traits (New York: Springer Nature), 245–283. 10.1007/978-1-0716-2205-6_9 35451779

[ref9] Desai S. V. Balasubramanian V. N. Fukatsu T. Ninomiya S. Guo W. (2019). Automatic estimation of heading date of paddy rice using deep learning. Plant Methods 15:76. doi: 10.1186/s13007-019-0457-1, PMID: 31338116 PMC6626381

[ref10] Fisher J. I. Mustard J. F. (2007). Cross-scalar satellite phenology from ground, Landsat, and MODIS data. Remote Sens. Environ. 109, 261–273. doi: 10.1016/j.rse.2007.01.004

[ref11] Fox J. Weisberg S. (2019). An R companion to applied regression. Thousand Oaks, CA: Sage.

[ref13] Ge H. X. Ma F. Li Z. W. Tan Z. Z. Du C. W. (2021). Improved accuracy of phenological detection in rice breeding by using ensemble models of machine learning based on UAV-RGB imagery. Remote Sens. 13:2678. doi: 10.3390/rs13142678

[ref14] Gitelson A. A. Gritz Y. Merzlyak M. N. (2003). Relationships between leaf chlorophyll content and spectral reflectance and algorithms for non-destructive chlorophyll assessment in higher plant leaves. J. Plant Physiol. 160, 271–282. doi: 10.1078/0176-1617-00887, PMID: 12749084

[ref15] Gitelson A. A. Vina A. Ciganda V. Rundquist D. C. Arkebauer T. J. (2005). Remote estimation of canopy chlorophyll content in crops. Geophys. Res. Lett. 32:L08403. doi: 10.1029/2005GL022688

[ref16] Guo W. Carroll M. E. Singh A. Swetnam T. L. Merchant N. Sarkar S. . (2021). UAS-based plant phenotyping for research and breeding applications. Plant Phenom. 2021:9840192. doi: 10.34133/2021/9840192 PMC8214361 34195621

[ref17] Guo Y. H. Chen S. Z. Fu Y. S. H. Xiao Y. Wu W. X. Wang H. X. . (2022). Comparison of multi-methods for identifying maize phenology using PhenoCams. Remote Sens. 14:244. doi: 10.3390/rs14020244, PMID: 39659294

[ref18] Han L. Yang G. J. Yang H. Xu B. Li Z. H. Yang X. D. (2018). Clustering field-based maize phenotyping of plant-height growth and canopy spectral dynamics using a UAV remote-sensing approach. Front. Plant Sci. 9:1638. doi: 10.3389/fpls.2018.01638, PMID: 30483291 PMC6244040

[ref19] Hastie T. Tibshirani R. Friedman J. (2009). The elements of statistical learning. New York: Springer.

[ref20] Heredia M. C. Kant J. Prodhan M. A. Dixit S. Wissuwa M. (2022). Breeding rice for a changing climate by improving adaptations to water saving technologies. Theor. Appl. Genet. 135, 17–33. doi: 10.1007/s00122-021-03899-8, PMID: 34218290

[ref21] Holman F. H. Riche A. B. Michalski A. Castle M. Wooster M. J. Hawkesford M. J. (2016). High throughput field phenotyping of wheat plant height and growth rate in field plot trials using UAV based remote sensing. Remote Sens. 8:8. doi: 10.3390/rs8121031, PMID: 39659294

[ref22] Hori K. Matsubara K. Yano M. (2016). Genetic control of flowering time in rice: integration of Mendelian genetics and genomics. Theor. Appl. Genet. 129, 2241–2252. doi: 10.1007/s00122-016-2773-4, PMID: 27695876

[ref23] Hori K. Nonoue Y. Ono N. Shibaya T. Ebana K. Matsubara K. . (2015). Genetic architecture of variation in heading date among Asian rice accessions. BMC Plant Biol. 15:115. doi: 10.1186/s12870-015-0501-x, PMID: 25953146 PMC4424449

[ref24] Horie T. Nakagawa H. Centeno H. Kropff M. (1995). “The rice crop simulation model SIMRIW and its testing,” in Modeling the impact of climate change on rice production in Asia, eds. Matthews R. B. Kropff M. J. Bachelet D. Laar H. H. Van. (Los Banos, Philippines: International Rice Research Institute), 51–66.

[ref26] Jiao Y. Q. Wang Y. H. Xue D. W. Wang J. Yan M. X. Liu G. F. . (2010). Regulation of OsSPL14 by OsmiR156 defines ideal plant architecture in rice. Nat. Genet. 42, 541–544. doi: 10.1038/ng.591, PMID: 20495565

[ref27] Kamiji Y. Yoshida H. Palta J. A. Sakuratani T. Shiraiwa T. (2011). N applications that increase plant N during panicle development are highly effective in increasing spikelet number in rice. Field Crop Res. 122, 242–247. doi: 10.1016/j.fcr.2011.03.016

[ref28] Khush G. S. (2013). Strategies for increasing the yield potential of cereals: case of rice as an example. Plant Breed. 132, 433–436. doi: 10.1111/pbr.1991

[ref29] Konietschke F. Placzek M. Schaarschmidt F. Hothorn L. A. (2015). Nparcomp: an R software package for nonparametric multiple comparisons and simultaneous confidence intervals. J. Stat. Softw. 64, 1–7. doi: 10.18637/jss.v064.i09

[ref30] Kronenberg L. Yates S. Boer M. P. Kirchgessner N. Walter A. Hund A. (2021). Temperature response of wheat affects final height and the timing of stem elongation under field conditions. J. Exp. Bot. 72, 700–717. doi: 10.1093/jxb/eraa471, PMID: 33057698 PMC7853599

[ref31] Li Z. K. Pinson S. R. M. Stansel J. W. Paterson A. H. (1998). Genetic dissection of the source-sink relationship affecting fecundity and yield in rice (*Oryza sativa* L.). Mol. Breed. 4, 419–426. doi: 10.1023/A:1009608128785

[ref32] Li B. Xu X. M. Han J. W. Zhang L. Bian C. S. Jin L. P. . (2019). The estimation of crop emergence in potatoes by UAV RGB imagery. Plant Methods 15:15. doi: 10.1186/s13007-019-0399-7, PMID: 30792752 PMC6371461

[ref33] Liu J. Zhan P. (2016). “The impacts of smoothing methods for time-series remote sensing data on crop phenology extraction”, in: 2016 IEEE international geoscience and remote sensing symposium (IGARSS).

[ref34] Lu X. Y. Zhou J. Yang R. Yan Z. Y. Lin Y. Y. Jiao J. . (2023). Automated rice phenology stage mapping using UAV images and deep learning. Drones 7:7. doi: 10.3390/drones7020083, PMID: 39659294

[ref35] Masjedi A. Crawford M. M. Carpenter N. R. Tuinstra M. R. (2020). Multi-temporal predictive modelling of Sorghum biomass using UAV-based hyperspectral and LiDAR data. Remote Sens. 12:3587. doi: 10.3390/rs12213587

[ref36] Ninomiya S. (2022). High-throughput field crop phenotyping: current status and challenges. Breed. Sci. 72, 3–18. doi: 10.1270/jsbbs.21069, PMID: 36045897 PMC8987842

[ref37] Ogawa D. Nonoue Y. Tsunematsu H. Kanno N. Yamamoto T. Yonemaru J. (2018a). Discovery of QTL alleles for grain shape in the Japan-MAGIC Rice population using haplotype information. G3 Genes Genomes Genet. 8, 3559–3565. doi: 10.1534/g3.118.200558, PMID: 30194091 PMC6222584

[ref38] Ogawa D. Sakamoto T. Tsunematsu H. Kanno N. Nonoue Y. Yonemaru J. I. (2021a). Haplotype analysis from unmanned aerial vehicle imagery of rice MAGIC population for the trait dissection of biomass and plant architecture. J. Exp. Bot. 72, 2371–2382. doi: 10.1093/jxb/eraa605, PMID: 33367626 PMC8006554

[ref39] Ogawa D. Sakamoto T. Tsunematsu H. Kanno N. Nonoue Y. Yonemaru J. I. (2021b). Remote-sensing-combined haplotype analysis using multi-parental advanced generation inter-cross lines reveals phenology QTLs for canopy height in rice. Front. Plant Sci. 12:715184. doi: 10.3389/fpls.2021.715184, PMID: 34721450 PMC8553969

[ref40] Ogawa D. Sakamoto T. Tsunematsu H. Yamamoto T. Kanno N. Nonoue Y. . (2019). Surveillance of panicle positions by unmanned aerial vehicle to reveal morphological features of rice. PLoS One 14:e0224386. doi: 10.1371/journal.pone.0224386, PMID: 31671163 PMC6822732

[ref41] Ogawa D. Yamamoto E. Ohtani T. Kanno N. Tsunematsu H. Nonoue Y. . (2018b). Haplotype-based allele mining in the Japan-MAGIC rice population. Sci. Rep. 8:8. doi: 10.1038/s41598-018-22657-3, PMID: 29531264 PMC5847589

[ref42] Ohsumi A. Takai T. Ida M. Yamamoto T. Arai-Sanoh Y. Yano M. . (2011). Evaluation of yield performance in rice near-isogenic lines with increased spikelet number. Field Crop Res. 120, 68–75. doi: 10.1016/j.fcr.2010.08.013

[ref43] Qiu Z. C. Xiang H. T. Ma F. Du C. W. (2020). Qualifications of rice growth indicators optimized at different growth stages using unmanned aerial vehicle digital imagery. Remote Sens. 12:3228. doi: 10.3390/rs12193228

[ref44] Reynolds D. Baret F. Welcker C. Bostrom A. Ball J. Cellini F. . (2019). What is cost-efficient phenotyping? Optimizing costs for different scenarios. Plant Sci. 282, 14–22. doi: 10.1016/j.plantsci.2018.06.015, PMID: 31003607

[ref45] Sakamoto T. (2020). Incorporating environmental variables into a MODIS-based crop yield estimation method for United States corn and soybeans through the use of a random forest regression algorithm. ISPRS J. Photogramm. Remote Sens. 160, 208–228. doi: 10.1016/j.isprsjprs.2019.12.012

[ref46] Sakamoto T. Gitelson A. A. Arkebauer T. J. (2013). MODIS-based corn grain yield estimation model incorporating crop phenology information. Remote Sens. Environ. 131, 215–231. doi: 10.1016/j.rse.2012.12.017

[ref47] Sakamoto T. Ogawa D. Hiura S. Iwasaki N. (2022). Alternative procedure to improve the positioning accuracy of orthomosaic images acquired with Agisoft Metashape and DJI P4 multispectral for crop growth observation. Photogramm. Eng. Remote. Sens. 88, 323–332. doi: 10.14358/PERS.21-00064R2

[ref48] Sakurai K. Toda Y. Hamazaki K. Ohmori Y. Yamasaki Y. Takahashi H. . (2023). Random regression for modeling soybean plant response to irrigation changes using time-series multispectral data. Front. Plant Sci. 14:1201806. doi: 10.3389/fpls.2023.1201806, PMID: 37476172 PMC10354427

[ref49] Shafiee S. Lied L. M. Burud I. Dieseth J. A. Alsheikh M. Lillemo M. (2021). Sequential forward selection and support vector regression in comparison to LASSO regression for spring wheat yield prediction based on UAV imagery. Comput. Electron. Agric. 183:106036. doi: 10.1016/j.compag.2021.106036

[ref50] Sheehy J. E. Dionora M. J. A. Mitchell P. L. (2001). Spikelet numbers, sink size and potential yield in rice. Field Crop Res. 71, 77–85. doi: 10.1016/S0378-4290(01)00145-9

[ref51] Shi Y. Y. Thomasson J. A. Murray S. C. Pugh N. A. Rooney W. L. Shafian S. . (2016). Unmanned aerial vehicles for high-throughput phenotyping and agronomic research. PLoS One 11:e0159781. doi: 10.1371/journal.pone.0159781, PMID: 27472222 PMC4966954

[ref52] Shinada H. Yamamoto T. Yamamoto E. Hori K. Yonemaru J. Matsuba S. . (2014). Historical changes in population structure during rice breeding programs in the northern limits of rice cultivation. Theor. Appl. Genet. 127, 995–1004. doi: 10.1007/s00122-014-2274-2, PMID: 24510168

[ref53] Son N. T. Chen C. F. Chang L. Y. Chen C. R. Sobue S. I. Minh V. Q. . (2016). A logistic-based method for rice monitoring from multi-temporal MODIS-Landsat fusion data. Eur. J. Remote Sens. 49, 39–56. doi: 10.5721/EuJRS20164903

[ref54] Taniguchi S. Sakamoto T. Imase R. Nonoue Y. Tsunematsu H. Goto A. . (2022). Prediction of heading date, culm length, and biomass from canopy-height-related parameters derived from time-series UAV observations of rice. Front. Plant Sci. 13:998803. doi: 10.3389/fpls.2022.998803, PMID: 36582650 PMC9792801

[ref55] Tao H. L. Feng H. K. Xu L. J. Miao M. K. Yang G. J. Yang X. D. . (2020). Estimation of the yield and plant height of winter wheat using UAV-based hyperspectral images. Sensors 20:1231. doi: 10.3390/s20041231, PMID: 32102358 PMC7070520

[ref56] Tester M. Langridge P. (2010). Breeding technologies to increase crop production in a changing world. Science 327, 818–822. doi: 10.1126/science.1183700, PMID: 20150489

[ref57] Tsukaguchi T. Kobayashi H. Fujihara Y. Chono S. (2022). Estimation of spikelet number per area by UAV-acquired vegetation index in rice (*Oryza sativa* L.). Plant Prod. Sci. 25, 20–29. doi: 10.1080/1343943X.2021.1943467

[ref58] Vicentini G. Biancucci M. Mineri L. Chirivi D. Giaume F. Miao Y. . (2023). Environmental control of rice flowering time. Plant Commun. 4:100610. doi: 10.1016/j.xplc.2023.100610, PMID: 37147799 PMC10504588

[ref59] Viña A. Gitelson A. A. Nguy-Robertson A. L. Peng Y. (2011). Comparison of different vegetation indices for the remote assessment of green leaf area index of crops. Remote Sens. Environ. 115, 3468–3478. doi: 10.1016/j.rse.2011.08.010

[ref60] Wan L. Cen H. Y. Zhu J. P. Zhang J. F. Zhu Y. M. Sun D. W. . (2020). Grain yield prediction of rice using multi-temporal UAV-based RGB and multispectral images and model transfer - a case study of small farmlands in the South of China. Agric. For. Meteorol. 291:108096. doi: 10.1016/j.agrformet.2020.108096

[ref61] Wang X. Q. Zhang R. Y. Song W. Han L. Liu X. L. Sun X. . (2019). Dynamic plant height QTL revealed in maize through remote sensing phenotyping using a high-throughput unmanned aerial vehicle (UAV). Sci. Rep. 9:3458. doi: 10.1038/s41598-019-39448-z 30837510 PMC6401315

[ref62] Westoby M. J. Brasington J. Glasser N. F. Hambrey M. J. Reynolds J. M. (2012). ‘Structure-from-motion’ photogrammetry: a low-cost, effective tool for geoscience applications. Geomorphology 179, 300–314. doi: 10.1016/j.geomorph.2012.08.021

[ref63] Xu W. C. Chen P. C. Zhan Y. L. Chen S. D. Zhang L. Lan Y. B. (2021). Cotton yield estimation model based on machine learning using time series UAV remote sensing data. Int. J. Appl. Earth Obs. Geoinf. 104:102511. doi: 10.1016/j.jag.2021.102511

[ref64] Xue J. R. Su B. F. (2017). Significant remote sensing vegetation indices: a review of developments and applications. J Sens 2017, 1–17. doi: 10.1155/2017/1353691

[ref65] Yang G. J. Liu J. G. Zhao C. J. Li Z. H. Huang Y. B. Yu H. Y. . (2017). Unmanned aerial vehicle remote sensing for field-based crop phenotyping: current status and perspectives. Front. Plant Sci. 8:1111. doi: 10.3389/fpls.2017.01111, PMID: 28713402 PMC5492853

[ref66] Yang X. Mustard J. F. Tang J. W. Xu H. (2012). Regional-scale phenology modeling based on meteorological records and remote sensing observations. J. Geophys. Res. Biogeosci. 117:G03029. doi: 10.1029/2012JG001977

[ref67] Yang Q. Shi L. S. Han J. Y. Chen Z. W. Yu J. (2022). A VI-based phenology adaptation approach for rice crop monitoring using UAV multispectral images. Field Crop Res. 277:108419. doi: 10.1016/j.fcr.2021.108419

[ref68] Yang Q. Shi L. S. Han J. Y. Yu J. Huang K. (2020). A near real-time deep learning approach for detecting rice phenology based on UAV images. Agric. For. Meteorol. 287:107938. doi: 10.1016/j.agrformet.2020.107938

[ref69] Yoshida H. Horie T. (2009). A process model for explaining genotypic and environmental variation in growth and yield of rice based on measured plant N accumulation. Field Crop Res. 113, 227–237. doi: 10.1016/j.fcr.2009.05.010

[ref70] Yoshida H. Horie T. Shiraiwa T. (2006). A model explaining genotypic and environmental variation of rice spikelet number per unit area measured by cross-locational experiments in Asia. Field Crop Res. 97, 337–343. doi: 10.1016/j.fcr.2005.11.004

