# Correction: Phenology analysis for trait prediction using UAVs in a MAGIC rice population with different transplanting protocols

**DOI:** 10.3389/frai.2025.1725594

**Published:** 2025-11-25

**Authors:** Shoji Taniguchi, Toshihiro Sakamoto, Haruki Nakamura, Yasunori Nonoue, Di Guan, Akari Fukuda, Hirofumi Fukuda, Kaede C. Wada, Takuro Ishii, Jun-Ichi Yonemaru, Daisuke Ogawa

**Affiliations:** 1 Research Center for Agricultural Information Technology, National Agricultural and Food Research Organization (NARO), Tokyo, Japan; 2 Institute for Agro-Environmental Sciences, NARO, Tsukuba, Japan; 3 Institute of Crop Science, NARO, Tsukuba, Japan

**Keywords:** rice, phenology, time-series analysis, MAGIC, UAV, remote sensing, transplanting protocol

In the published article, there was an error in Figure 9B and Figure 10B as published. The CH and CIg parameter data for the 2023 dataset were incorrectly labeled: the transplanting protocols Regular (R) and Delayed (D) were mistakenly swapped. The corrected Figure 9B and Figure 10B appear below.

**Figure 9:**
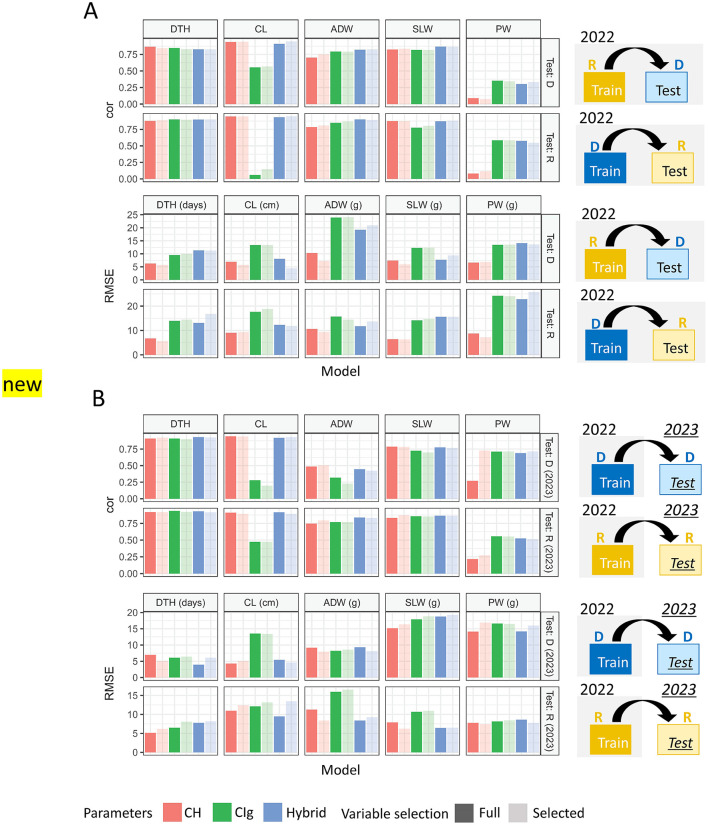


**Figure 10:**
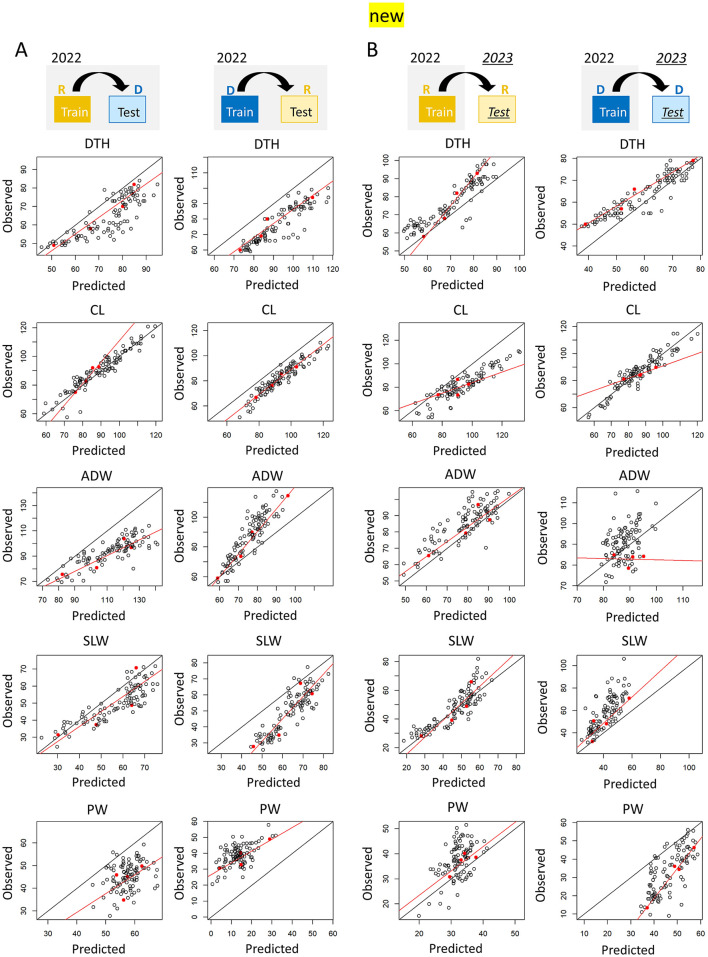


In the published article, there was an error in Supplementary Table 13, Table 15, which is in Supplementary Table 1. The CH and CIg parameter data for the 2023 dataset were incorrectly labeled: the transplanting protocols Regular (R) and Delayed (D) were mistakenly swapped. The correct material statement appears in the updated Supplementary Table 1.

In the published article, there was an error in Supplementary Figure 8. The CH and CIg parameter data for the 2023 dataset were incorrectly labeled; the transplanting protocols Regular (R) and Delayed (D) were mistakenly swapped. Supplementary Presentation 1 has been updated.

In the published article, there was an error in Supplementary Data Sheet 1. The CH and CIg parameter labels for the 2023 dataset have been revised. This dataset was used for reanalysis and supports the updated results presented in the corrected figures, tables and text. The correct material statement appears in the updated Supplementary Data Sheet 1.

In the published article, there was an error. The CH and CIg parameter data for the 2023 dataset were incorrectly labeled: the transplanting protocols Regular (R) and Delayed (D) were mistakenly swapped.

A correction has been made to **Results**, *3.5 Calibration of training and test data obtained under different protocols*, Paragraph 1. This text previously stated:

“The calibration also reduced the RMSE values for the prediction of ADW, SLW, and PW (Supplementary Table 14). In terms of type-2 model robustness, calibration reduced the RMSE values for CL, ADW, PW, and DTH (Supplementary Table 15). There were, however, two cases where calibration resulted in a larger RMSE: the prediction of CL under the delayed transplanting protocol (type-1 model robustness) and the prediction of SLW of the delayed protocol in 2023 (type-2 model robustness). In these two cases, the phenotypic data of the four parental cultivars did not cover the full range of phenotypic variance of the JAM2 lines.”

The corrected sentence appears below:

“The calibration also reduced the RMSE values for the prediction of ADW, SLW, and PW (Supplementary Table 14). There was one case where calibration resulted in a larger RMSE: the prediction of CL under the delayed transplanting protocol. In terms of type-2 model robustness, calibration reduced the RMSE values for PW (Supplementary Table 15), but the calibration did not always work well for CL, ADW, DTH and SLW. In these cases, the phenotypic data of the four parental cultivars did not cover the full range of phenotypic variance of the JAM2 lines.”

A correction has been made to **Discussion**, Paragraph 6. The sentence previously stated:

“Only in the two cases did the calibration not work well. This may be because the phenotypic data …”

The corrected sentence appears below:

“The calibration did not work well for some cases. This may be because the phenotypic data …”

The original article has been updated.

