# Supplementary material for: Phenology analysis for trait prediction using UAVs in a MAGIC rice population with different transplanting protocols
Source: Front Artif Intell. 2025 Jan 23;7:1477637. doi: 10.3389/frai.2024.1477637 (PMC11799559; doi:10.3389/frai.2024.1477637)
Supplement: Supplementary file 3 [file Presentation_1.pptx]

## Slide 1
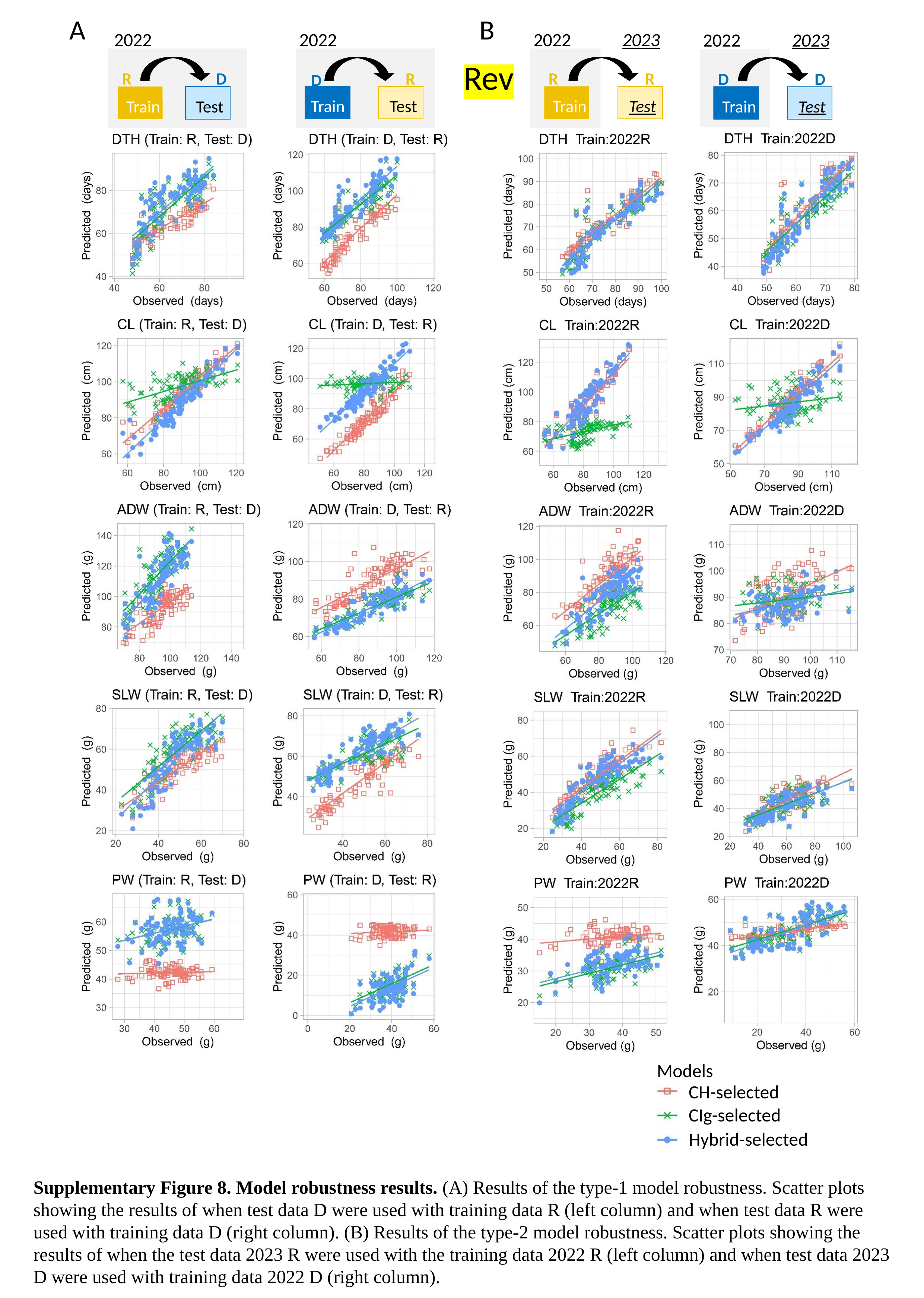

A
B
2022
2022
2022
2023
2022
2023
Rev
R
R
D
R
R
D
D
D
Train
Test
Train
Test
Train
Train
Test
Test
Models
CH-selected
CIg-selected
Hybrid-selected
Supplementary Figure 8. Model robustness results. (A) Results of the type-1 model robustness. Scatter plots showing the results of when test data D were used with training data R (left column) and when test data R were used with training data D (right column). (B) Results of the type-2 model robustness. Scatter plots showing the results of when the test data 2023 R were used with the training data 2022 R (left column) and when test data 2023 D were used with training data 2022 D (right column).
